# Genomic binding and regulation of gene expression by the thyroid carcinoma-associated PAX8-PPARG fusion protein

**DOI:** 10.18632/oncotarget.6340

**Published:** 2015-11-16

**Authors:** Yanxiao Zhang, Jingcheng Yu, Chee Lee, Bin Xu, Maureen A. Sartor, Ronald J. Koenig

**Affiliations:** ^1^ Department of Computational Medicine and Bioinformatics, University of Michigan, Ann Arbor, MI, USA; ^2^ Division of Metabolism, Endocrinology and Diabetes, Department of Internal Medicine, University of Michigan, Ann Arbor, MI, USA

**Keywords:** peroxisome proliferator-activated receptor gamma, follicular thyroid cancer, pioglitazone, differentiation, gene fusion

## Abstract

A chromosomal translocation results in production of an oncogenic PAX8-PPARG fusion protein (PPFP) in thyroid carcinomas. PAX8 is a thyroid transcription factor, and PPARG is a transcription factor that plays important roles in adipocytes and macrophages. PPFP retains the DNA binding domains of both proteins; however, the genomic binding sites of PPFP have not been identified, and only limited data exist to characterize gene expression in PPFP thyroid carcinomas. Therefore, the oncogenic function of PPFP is poorly understood. We expressed PPFP in PCCL3 rat thyroid cells and used ChIP-seq to identify PPFP genomic binding sites (PPFP peaks) and RNA-seq to characterize PPFP-dependent gene expression. PPFP peaks (~20,000) include known PAX8 and PPARG binding sites and are enriched with both motifs, indicating that both DNA binding domains are functional. PPFP binds to and regulates many genes involved in cancer-related processes. In PCCL3 thyroid cells, PPFP binds to adipocyte PPARG target genes in preference to macrophage PPARG target genes, consistent with the pro-adipogenic nature of PPFP and its ligand pioglitazone in thyroid cells. PPFP induces oxidative stress in thyroid cells, and pioglitazone increases susceptibility to further oxidative stress. Our data highlight the complexity of PPFP as a transcription factor and the numerous ways that it regulates thyroid oncogenesis.

## INTRODUCTION

Thyroid carcinoma is the most common endocrine malignancy, and its incidence has increased nearly 3-fold since 1990 [1, 2]. The majority of thyroid carcinomas contain one of a small number of driver mutations, such as *BRAF* or *RAS* mutations, gene fusions involving *RET*, or gene fusions between *PAX8* and *PPARG* (reviewed in [3]). The *PAX8*- *peroxisome proliferator-activated receptor gamma* (*PPARG)* gene fusion is a consequence of a translocation between chromosomes 2 and 3, and is found in ~30% of follicular thyroid carcinomas and ~5% of follicular variant papillary carcinomas. The resulting PAX8-PPARG fusion protein (PPFP) is unusual in that it is the fusion of two transcription factors and it retains the DNA binding domains (DBDs) of both parent proteins [4]. Thus, at least in principle, PPFP should be capable of binding to PAX8 and PPARG response elements and potentially regulating target genes of both transcription factors. However, no data exist to define the genomic binding sites of PPFP, and the largest study characterizing global gene expression patterns in human PPFP carcinomas consisted of only 7 cases [5]. Given these limited data, the mechanism of oncogenesis is poorly understood (reviewed in [6]).

PAX8 is a member of the paired box family of transcription factors and is essential for thyroid gland development [7, 8]. In the mature thyroid, PAX8 drives the expression of numerous thyroid-specific genes [8]. PPARG is a member of the nuclear receptor family of transcription factors. It has no identified role in the normal thyroid and is expressed at extremely low levels in that organ. PPARG is the master regulator of adipogenesis [9], and also plays an important role in macrophage development, where it promotes an anti-inflammatory phenotype [10]. Synthetic agonist ligands for PPARG such as pioglitazone are insulin sensitizers and hence are used to treat type 2 diabetes. PPARG ligands also are ligands for PPFP. In a mouse model of PPFP thyroid carcinoma, pioglitazone was highly therapeutic, greatly shrinking thyroid size and preventing metastatic disease [11]. Pioglitazone was strongly pro-adipogenic in these murine thyroid tumors, converting the thyroid cells into lipid-laden adipocyte-like cells. Although this indicates that PPFP is strongly PPARG-like in the presence of pioglitazone, the mechanism underlying the therapeutic efficacy of pioglitazone in this mouse model of PPFP thyroid carcinoma is not known.

There are no existing cell lines from PPFP thyroid carcinomas. However, PPFP has been stably expressed in the PCCL3 rat thyroid cell line at a level comparable to that in human thyroid cancers, herein denoted PPFP cells [12]. PPFP expression confers upon PCCL3 cells an increased ability to invade through Matrigel and to form colonies in soft agar, both signs of cellular transformation [12]. Thus, PPFP cells are a useful cell culture model to study PPFP-dependent oncogenesis, and potentially, the response to pioglitazone. PCCL3 cells also have been used to create cell culture models of thyroid carcinomas caused by oncogenic driver mutations in *BRAF* [13] and *RAS* [14], and *RET* gene fusions [15].

Here, we have used RNA deep sequencing (RNA-seq) to study the gene expression of PPFP cells versus control empty vector (EV) cells, cultured with and without pioglitazone. We also performed chromatin immunoprecipitation-deep sequencing (ChIP-seq) to identify the PPFP binding sites within the PCCL3 cell genome, and integrated the results with the gene expression data and publicly-available PAX8 and PPARG ChIP-seq data. The results provide novel insights into the transcriptional regulatory activity of PPFP, its oncogenic actions, and the response to pioglitazone.

## RESULTS

### Overview of genes regulated by PPFP in the absence and presence of pioglitazone

**Figure 1:**
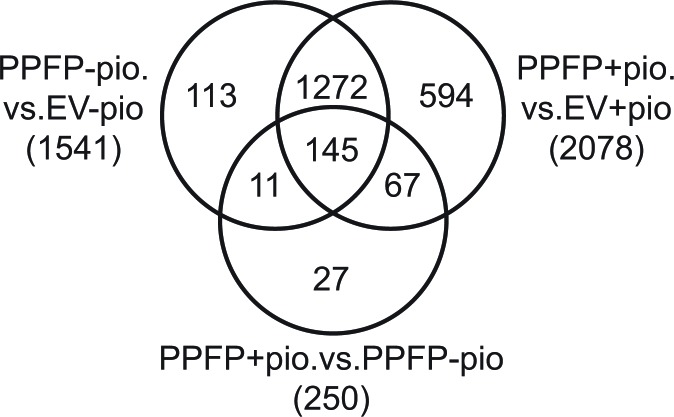
Venn diagram illustrating the overlap of genes regulated by PPFP in comparisons of PPFP and EV cells cultured with and without pioglitazone The total number of differentially expressed genes for each comparison is shown in parentheses, using FDR<.05 and fold change >2 as cut-offs.

An RNA-seq analysis was performed on RNA from PPFP cells versus EV cells treated with or without pioglitazone. PPFP regulated the expression of 1541 genes (628 up, 913 down) in the comparison of PPFP cells versus EV cells without pioglitazone (FDR <0.05 and fold change >2). When both cell lines were cultured with pioglitazone, slightly more genes were differentially expressed (2078; 877 up, 1201 down). In a comparison of PPFP cells cultured with versus without pioglitazone, 250 genes were differentially expressed (95 up, 155 down). The differentially expressed genes in all of these comparisons are highly overlapping (Figure 1). In contrast, there were no differentially expressed genes in EV cells cultured with versus without pioglitazone, consistent with the very low expression level of endogenous PPARG in thyroid cells and the specificity of pioglitazone. Figure 1 shows that 156 of the 250 genes differentially expressed in PPFP cells cultured with versus without pioglitazone also are differentially expressed in PPFP cells versus EV cells cultured without pioglitazone. The PPFP and pioglitazone-induced changes are in the same direction for 130 (83%) of these 156 genes (48 up, 82 down), indicating that pioglitazone reinforces most of the PPFP-induced changes. However, for 26 genes (17%), the changes were in opposite directions such that pioglitazone partially or completely reversed the effects of PPFP.

### PPFP regulates processes related to oncogenesis

**Figure 2:**
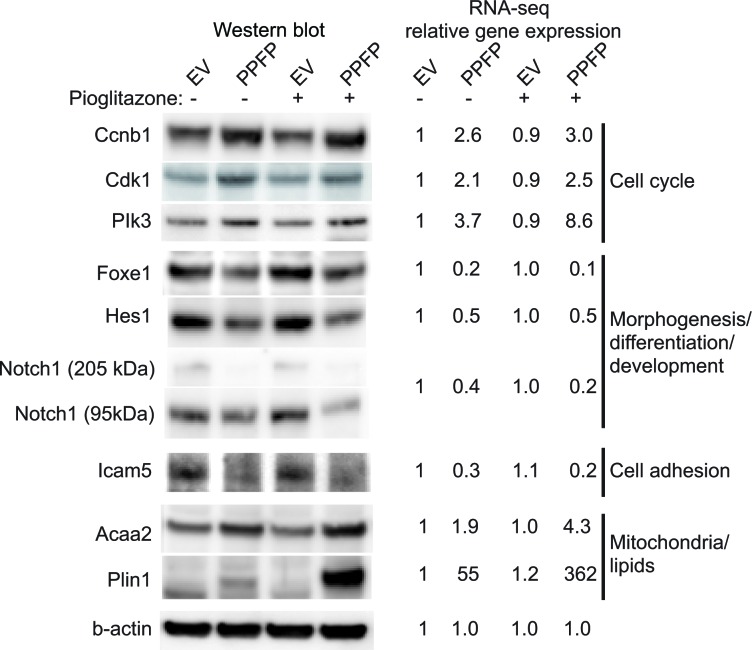
Western blot analysis and RNA-seq expression data of selected genes in PPFP and EV cells cultured without and with pioglitazone RNA-seq data are normalized relative to EV cells cultured without pioglitazone. The genes are organized by concepts related to the gene set names with which they are associated, as described in Results. Although grouped together in one figure, the expression of these proteins without and with pioglitazone is presented at several different places within Results.

**Figure 3:**
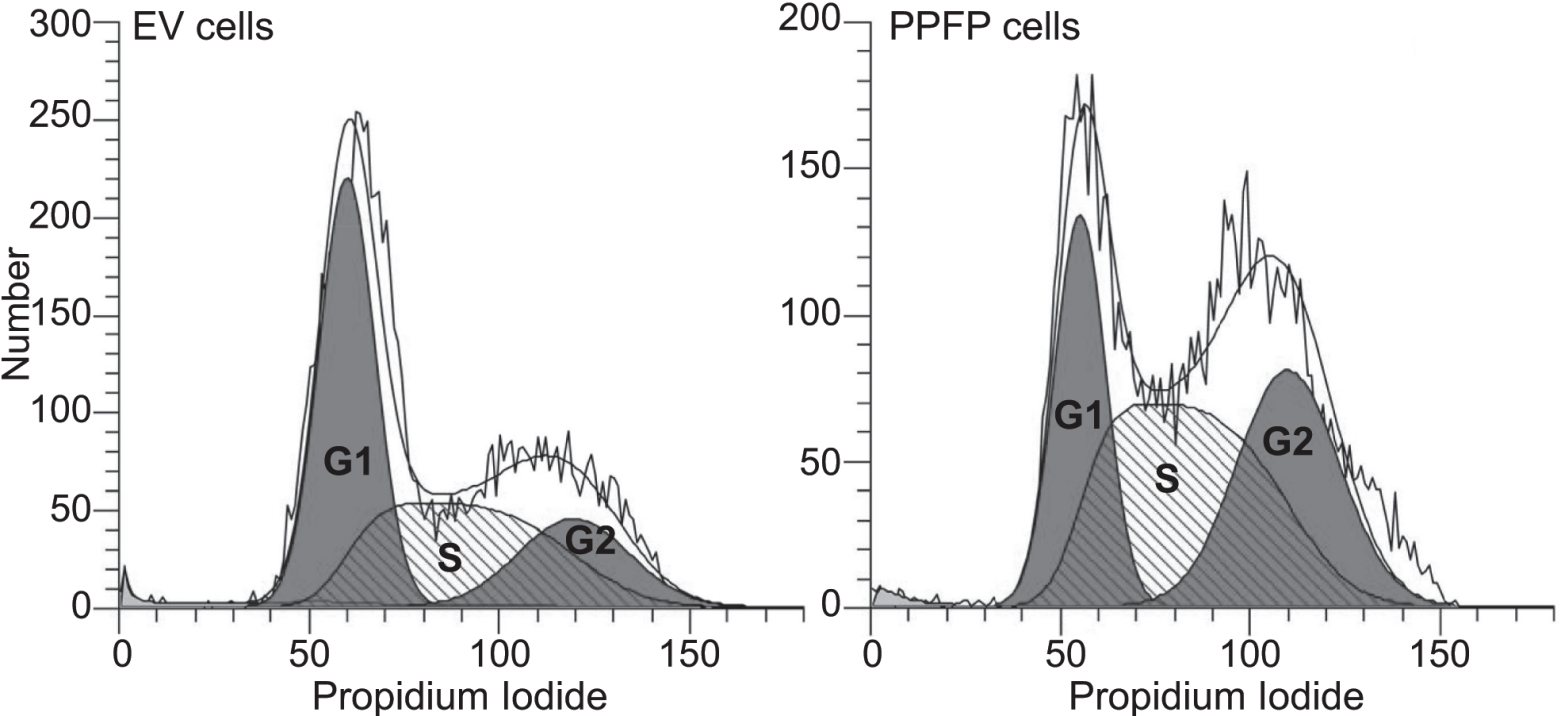
DNA content analysis of EV and PPFP cells Cells were fixed, stained with propidium iodide and analyzed by flow cytometry using ModFit LT version 4.1 software. The graphs show the histograms and the derived areas of the G1, S and G2/M (labeled G2) phases of the cell cycle. These are quantified for EV cells as G1 45%, S 36% and G2/M 19%; and for PPFP cells, G1 26%, S 43% and G2/M 31% of cells.

Gene expression changes in PPFP cells versus EV cells in the absence of pioglitazone potentially are relevant to the oncogenic actions of PPFP. We subjected this comparison to a functional enrichment analysis using Gene Ontology (GO) terms and KEGG pathways [16, 17]. We identified 162 enriched gene sets (FDR<0.05), 55 of which were induced by PPFP and 107 repressed. The 15 induced and 15 repressed gene sets with the lowest q-values are shown in Table 1, and the full list is provided in Supplemental Table S1. Many of the induced gene sets involve processes directly related to cancer biology. For example, gene sets related to the cell cycle include *MCM complex*, *deoxyribonucleotide biosynthetic process*, *DNA replication*, and others. Three cell cycle-related genes within these gene sets (*Ccnb1*, *Cdk1* and *Plk3*) were investigated further and were also found to be induced at the protein level by PPFP (Figure 2). Consistent with the enrichment of cell cycle-related gene sets, cellular DNA content analysis by flow cytometry demonstrated that a greater fraction of PPFP cells than EV cells are in the S and G2/M phases of the cell cycle, and a lesser fraction are in G1 (Figure 3).

Other highly significant cancer-related processes were related to proteasome/protein folding, immune function and oxidative stress (Table 1 and Supplemental Table S1). Gene sets related to mitochondria/lipids also were enriched, consistent with PPARG-like activity of PPFP. We confirmed the induction by PPFP of two such PPARG target genes, *Acaa2* and *Plin1*, at the protein level (Figure 2).

Twenty-two of the 107 repressed gene sets contain the word *morphogenesis, differentiation* or *development* (Tables 1 and S1), consistent with the expectation that, as an oncogene, PPFP enforces a less differentiated state. The repressed genes include several involved in thyroid differentiation, including *Fgfr2* [18], *Hhex* [19], *Foxe1* [20], *Hes1* [21] *and Notch1* [22], the latter three of which were confirmed at the protein level (Figure 2). The repressed gene sets also include *cell adhesion*, *extracellular matrix*, and several related terms. Repressing these gene sets could facilitate invasion and metastases. We confirmed PPFP-dependent repression of the cell adhesion protein Icam5 at the protein level (Figure 2).

**Table 1:** Fifteen induced and 15 repressed gene sets with the lowest q-values in PPFP cells versus EV cells cultured without pioglitazone

Concept.ID	Concept.name	*p*-value	*q*-value	Status PPFP *vs* EV
GO:0071346	cellular response to interferon-gamma	6.17E-07	1.37E-04	up
GO:0030529	ribonucleoprotein complex	8.09E-06	1.71E-04	up
GO:0005740	mitochondrial envelope	5.07E-05	6.49E-04	up
GO:0005811	lipid particle	8.27E-05	8.87E-04	up
GO:0046689	response to mercury ion	1.72E-05	1.05E-03	up
GO:0005730	nucleolus	2.08E-04	2.04E-03	up
GO:0004364	glutathione transferase activity	3.24E-05	2.25E-03	up
rno00480	Glutathione metabolism	1.29E-05	2.48E-03	up
GO:0042555	MCM complex	2.92E-04	2.54E-03	up
GO:0071219	cellular response to molecule of bacterial origin	6.99E-05	2.75E-03	up
GO:0071398	cellular response to fatty acid	1.02E-04	3.35E-03	up
GO:0006396	RNA processing	1.22E-04	3.56E-03	up
GO:0071384	cellular response to corticosteroid stimulus	1.23E-04	3.56E-03	up
GO:0000502	proteasome complex	5.06E-04	3.95E-03	up
GO:0006457	protein folding	2.13E-04	5.08E-03	up
GO:0008021	synaptic vesicle	1.36E-14	6.25E-12	down
GO:0007267	cell-cell signaling	1.98E-09	7.03E-06	down
GO:0004888	transmembrane signaling receptor activity	3.96E-08	2.75E-05	down
GO:0048858	cell projection morphogenesis	3.74E-08	2.95E-05	down
GO:0048730	epidermis morphogenesis	4.75E-08	2.95E-05	down
GO:0044306	neuron projection terminus	1.12E-06	3.45E-05	down
GO:0050808	synapse organization	1.10E-07	4.87E-05	down
GO:0048667	cell morphogenesis involved in neuron differentiation	1.38E-07	4.92E-05	down
GO:0004872	receptor activity	1.56E-07	5.40E-05	down
GO:0043534	blood vessel endothelial cell migration	2.87E-07	8.80E-05	down
GO:1901342	regulation of vasculature development	5.06E-07	1.28E-04	down
GO:0045995	regulation of embryonic development	1.15E-06	1.77E-04	down
GO:0090288	negative regulation of cellular response to growth factor stimulus	2.02E-06	2.55E-04	down
GO:0007167	enzyme linked receptor protein signaling pathway	2.91E-06	3.13E-04	down
GO:0051960	regulation of nervous system development	3.10E-06	3.24E-04	down

### PPFP can induce or repress PAX8-regulated genes

PAX8 induces thyroid-specific genes such as *Tg*, but only limited data exist to define PAX8-responsive genes more broadly. In a previous publication [23], siRNA knockdown of PAX8 in PCCL3 cells yielded 601 differentially expressed genes that also were tested in our data set. In general the magnitude of change was modest in the siRNA experiment, with 296 genes showing a fold change >1.2 (siPAX8 induced 175 genes and repressed 121). We determined what fraction of these siPAX8-responsive genes was differentially expressed in PPFP cells versus EV cells cultured without pioglitazone (using FDR<0.05 and fold change >1.5 as cut-offs). As shown in Table 2, slightly more than half of siPAX8-regulated genes are regulated by PPFP, and the direction of regulation is discordant about 2/3 of the time (*p* = 0.00015 for observing this level of discordance by chance; Fisher's exact test). Since induction by siPAX8 implies repression by PAX8 and vice versa, the data indicate that PAX8 and PPFP regulate gene expression in the same direction for ~2/3 of the genes, and in opposite directions for ~1/3.

**Table 2:** Regulation of PAX8-responsive genes by PPFP

	PPFP induces	PPFP represses	PPFP no change	Total
siPAX8 induces	32 (18%)	62 (35%)	81 (46%)	175 (100%)
siPAX8 represses	47 (39%)	24 (20%)	50 (41%)	121 (100%)
Total	79	86	131	296

### PPFP regulates genes related to fatty acid metabolism and mitochondrial function, especially in the presence of pioglitazone

The 55 induced gene sets in the comparison of PPFP cells versus EV cells in the absence of pioglitazone include several related to mitochondria, fatty acids and lipids, such as *mitochondrial envelope*, *lipid particle*, and *cellular response to fatty acid* (Tables 1 and S1). The induced genes in these gene sets include adipocyte PPARG target genes such as *Acaa2* and *Plin1*, demonstrating that PPFP is PPARG-like on a subset of target genes. This is consistent with the fact that several PPARG target genes have been shown to be induced in human PPFP thyroid carcinomas [5]. However, the PPARG-like activity of PPFP is much more striking in the presence of pioglitazone. We found that 117 gene sets are enriched in the comparison of PPFP cells cultured with versus without pioglitazone (52 induced, 65 repressed) (Supplemental Table S2). The 10 most significant gene sets are all induced by pioglitazone, and all relate to fatty acid metabolism and PPAR activity (Table 3). Among the PPARG target genes in these gene sets, we confirmed the inductions of *Acaa2* and *Plin1* at the protein level, as noted previously (Figure 2).

**Table 3:** The 10 induced gene sets with the lowest q-values in the comparison of PPFP cells cultured with versus without pioglitazone all relate to fatty acid metabolism, mitochondria and PPAR activity

Gene set ID	Description	*q*-value
GO:0009062	fatty acid catabolic process	7.00E-11
GO:0019395	fatty acid oxidation	1.53E-10
GO:0004091	carboxylesterase activity	1.93E-09
rno03320	PPAR signaling pathway	4.55E-08
GO:0006637	acyl-CoA metabolic process	2.15E-07
GO:0006641	triglyceride metabolic process	4.81E-07
GO:0005777	peroxisome	5.61E-07
GO:0005740	mitochondrial envelope	5.96E-07
GO:0005759	mitochondrial matrix	1.03E-06
GO:0071398	cellular response to fatty acid	2.74E-06

### Overview of the PPFP cistrome

**Figure 4:**
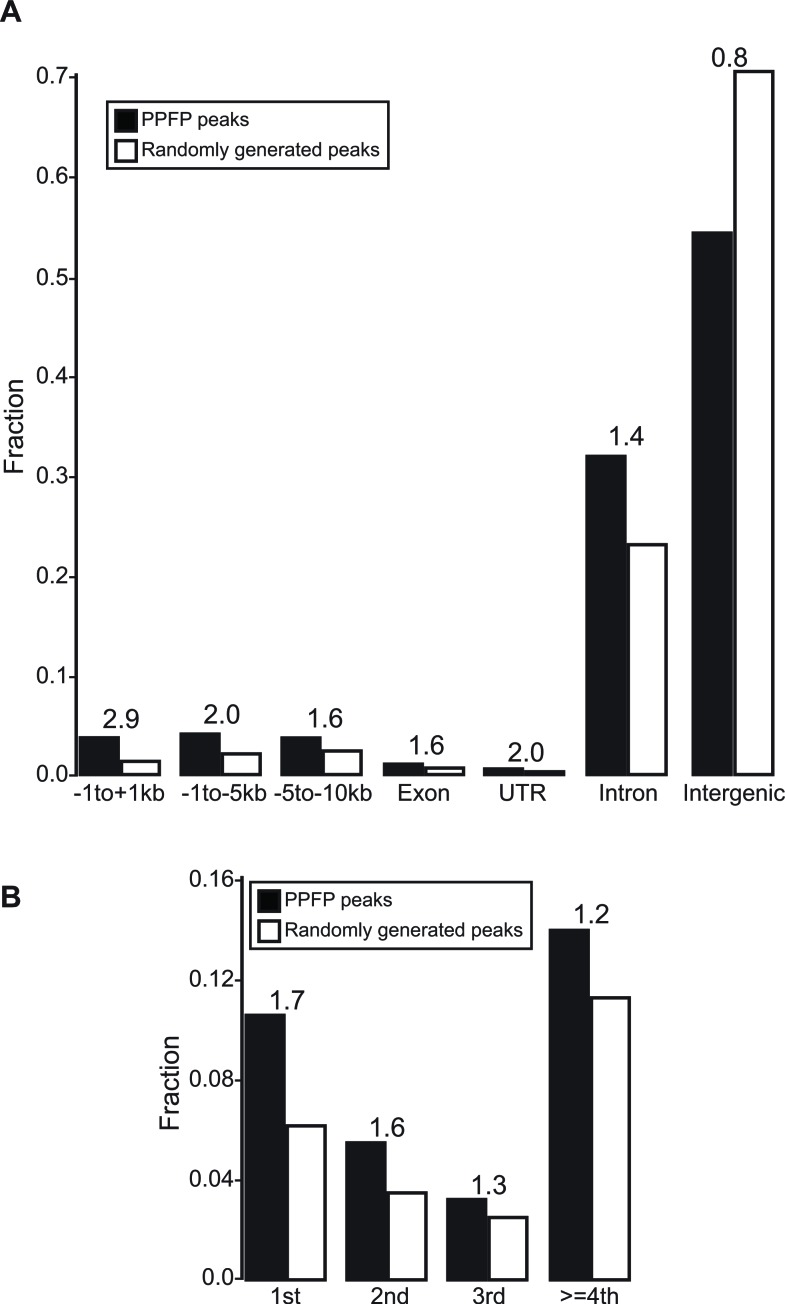
Annotation of PPFP peaks versus randomly generated peaks, relative to genic and intergenic regions **A.** Peaks were assigned to one region only with the prioritization going from left to right. **B.** The intron group of A is divided into individual introns. The numbers above the bars indicate the ratios of PPFP to randomly generated peaks.

We performed ChIP-seq analysis on PPFP to begin to understand the DNA binding properties of PPFP and the genes it is likely to regulate through direct interactions. Using an FDR<.05, we identified 20,277 PPFP peaks in the PCCL3 cell genome. As has been found previously for PAX8 [23] and PPARG [24], most PPFP peaks are intergenic. However, we observed an enrichment of PPFP peaks in genic regions, and most strikingly within 1 kb of transcription start sites (TSS's) (2.9-fold enriched) (Figure 4). PPFP peaks also are enriched 2-fold from −5 to −1 kb of TSS's and 1.7-fold in first introns, and are under-represented (0.8-fold) in intergenic regions.

**Figure 5:**
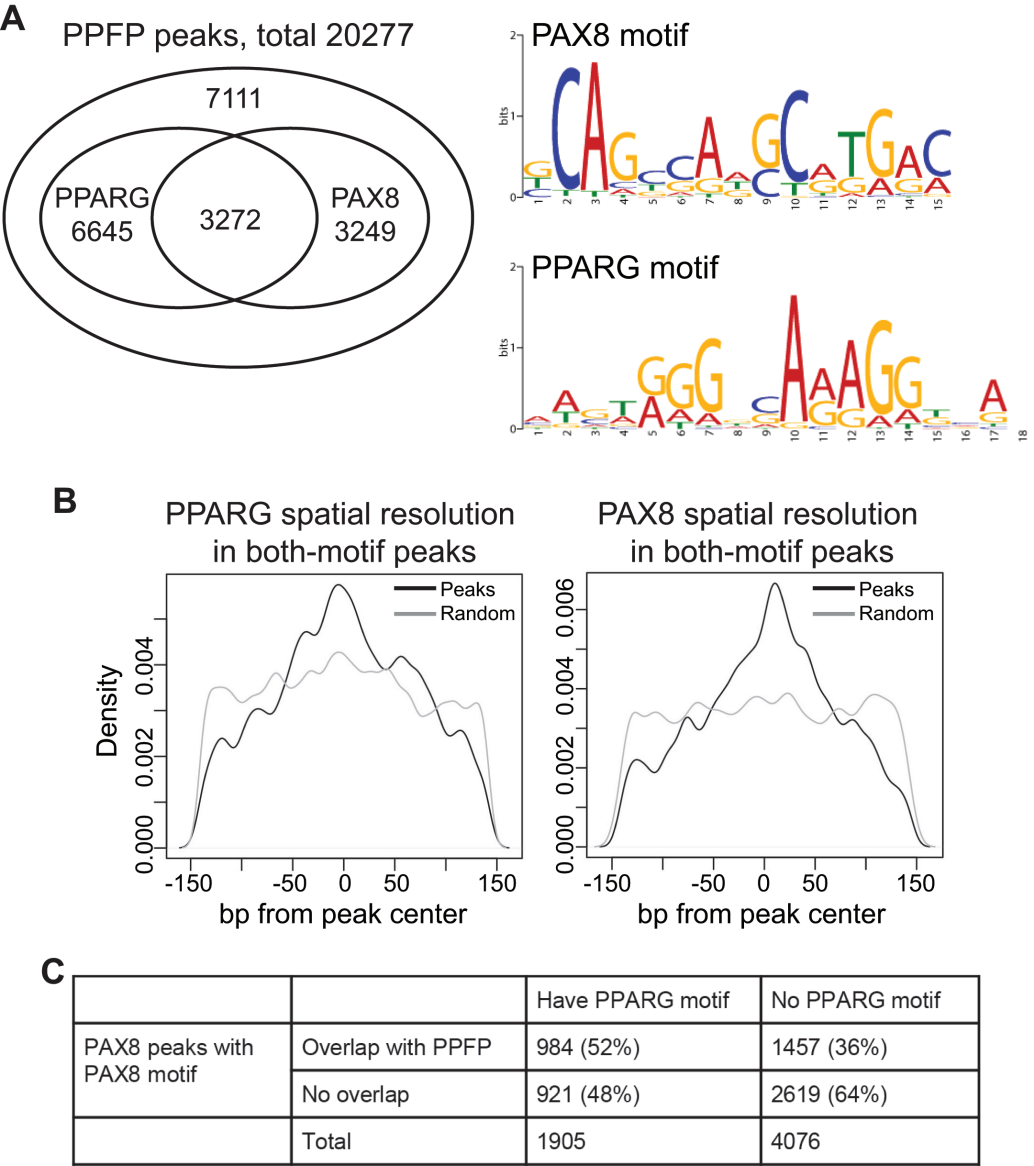
PPFP peaks contain PAX8 and/or PPARG motifs **A.** Venn diagram showing the overlap of PAX8 and PPARG motifs within PPFP peaks, and the logos for PAX8 and PPARG motifs. **B.** Spatial resolution analysis showing that both the PAX8 and PPARG motifs are centered in the PPFP peaks that contain both motifs (black lines). The grey lines show the flat distribution of each motif in randomly sampled 300bp regions across genome, serving as negative controls. **C.** The peaks with PAX8 motifs identified in a previously published [23] PAX8 ChIP-seq analysis of PCCL3 cells were divided into those that overlap or not with the PPFP peaks identified in this study. These were then subdivided into peaks with or without PPARG motifs. PPARG motifs are enriched in the PAX8 peaks that overlap with PPFP peaks, *p* < 2.2e-16, Fisher's exact test.

Since PPFP contains DBDs from both PAX8 and PPARG, in principle it could bind to the DNA motifs recognized by both transcription factors. This is what was observed, as we identified the PPARG and PAX8 motifs *de novo* as the top two most overrepresented sequences within the peak regions using HOMER [25]. Overall, 65% of PPFP peaks contain a PPARG motif and/or a PAX8 motif, and these partially overlap (Figure 5A). An unexpected finding was that 50% of the peaks with a PAX8 motif also contain a PPARG motif. This is interesting because a much lower rate of PPARG motifs would be expected near PAX8 motifs if PPFP uses only one of the two DBDs for every binding site. To investigate if the co-localizations of the two motifs were due to false-positive matches of the motif position weight matrices to the sequences, we examined the motif locations relative to the peak centers. The results show that both the PAX8 and PPARG motifs are centered within these PPFP peaks, as would be expected if both are functionally relevant to DNA binding (Figure 5B). This suggests that PPFP prefers to bind to the subset of PAX8 motifs that have nearby PPARG motifs. To evaluate this further, we took advantage of the fact that a ChIP-seq analysis has been published for PAX8 in PCCL3 cells [23]. We first filtered the PAX8 peaks by whether they contained at least one PAX8 motif, to remove potential false positive peaks that could confound the analysis. We then asked what fraction of the PAX8 peaks that contain a PAX8 motif and do or do not overlap with our PPFP peaks also contain a PPARG motif. As shown in Figure 5C, PPARG motifs are enriched in the PAX8 peaks to which PPFP also binds (odds ratio=1.9, *p*-value<2.2e-16, Fisher's exact test), confirming that PPFP preferentially binds to the subset of PAX8 peaks that also contain a PPARG motif.

### Overview of genes and gene sets containing PPFP peaks

PPFP peaks were found to encompass a number of known functional response elements in classic PAX8 and PPARG responsive genes. For example, PPFP peaks encompass the PAX8 response element in the *Tg* promoter [26] and the PPARG response element in the *Aqp7* promoter [27].

One hundred sixty eight GO terms were identified as enriched with PPFP peaks (FDR<0.05), after associating peaks with the gene having the nearest TSS (Supplemental Table S3). Nine of the 15 GO terms with the lowest *q*-values are related to immune function, development/differentiation, or lipid metabolism, and the GO terms in the full list include additional cancer-related concepts such as *negative regulation of programmed cell death*, *regulation of cell migration*, *G1/S transition of mitotic cell cycle*, and *Wnt receptor signaling pathway*.

In subsequent analyses, we focused on gene sets that are both enriched in the ChIP-seq analysis and enriched among the differentially expressed genes by RNA-seq analysis. Eight such gene sets were induced in PPFP cells versus EV cells cultured without pioglitazone, and 13 were repressed (Table 4). Seven of the 8 induced gene sets relate to mitochondria, and include direct PPARG target genes such as *Plin1*. The one additional induced gene set, *G1/S transition of mitotic cell cycle*, includes genes such as *Plk3* that promote progression through the cell cycle and cell division. The inductions of *Plin1* and *Plk3* were confirmed at the protein level, as noted previously (Figure 2).

In contrast, the 13 repressed gene sets relate mostly to protein signaling (4 gene sets), morphogenesis/development/differentiation (4 gene sets), and cell communication/extracellular matrix/adhesion (4 gene sets). The repressed genes in these gene sets include the thyroid development genes *Fgfr2* [18] and *Hhex* [19].

**Table 4:** Gene sets enriched in PPFP peaks by ChIP-seq analysis and differentially expressed in PPFP cells versus EV cells cultured without pioglitazone

Gene set ID	Description	*q*-value ChIP	*q*-value RNA-seq PPFP *vs* EV without pioglitazone	Status PPFP *vs* EV
GO:0005740	mitochondrial envelope	0.024	6.49E-04	induced
GO:0044429	mitochondrial part	8.30E-04	7.19E-04	induced
GO:0005743	mitochondrial inner membrane	0.029	7.19E-04	induced
GO:0031966	mitochondrial membrane	0.029	8.51E-04	induced
GO:0005811	lipid particle	0.0088	8.87E-04	induced
GO:0019866	organelle inner membrane	0.024	9.99E-04	induced
GO:0019915	lipid storage	0.030	0.010	induced
GO:0000082	G1/S transition of mitotic cell cycle	0.010	0.042	induced
GO:0007167	enzyme linked receptor protein signaling pathway	3.50E-04	3.13E-04	repressed
GO:0007169	transmembrane receptor protein tyrosine kinase signaling pathway	0.011	0.0016	repressed
GO:0022603	regulation of anatomical structure morphogenesis	7.16E-04	0.0019	repressed
GO:0010648	negative regulation of cell communication	6.59E-04	0.0028	repressed
GO:0045664	regulation of neuron differentiation	0.035	0.0053	repressed
GO:0023057	negative regulation of signaling	0.0014	0.0072	repressed
GO:0009968	negative regulation of signal transduction	3.08E-04	0.0080	repressed
GO:0031012	extracellular matrix	0.028	0.0081	repressed
GO:0035295	tube development	0.034	0.014	repressed
GO:0050867	positive regulation of cell activation	0.049	0.035	repressed
GO:0030155	regulation of cell adhesion	0.048	0.040	repressed
GO:0016331	morphogenesis of embryonic epithelium	0.042	0.043	repressed
GO:0005539	glycosaminoglycan binding	0.022	0.048	repressed

### PPFP functions through its PAX8 DBD to repress gene sets when bound at a distance from transcription start sites, but to induce gene sets when bound close to transcriptional start sites

We classified PPFP peaks as to whether they contain a PAX8 motif or a PPARG motif, and whether they are ≤10kb from a TSS or >10kb upstream from a TSS. We then performed gene set enrichment analyses on these 4 groups of PPFP peaks using ChIP-Enrich [28] and compared the results with the RNA-seq analysis of PPFP cells versus EV cells without pioglitazone.

Analysis of PPFP peaks with PAX8 motifs >10kb upstream from TSS's yielded no gene sets that were enriched with ChIP-seq peaks and induced by PPFP. However, 16 gene sets were enriched and repressed by PPFP, encompassing 59 unique genes. This suggests that, when regulating gene sets through PAX8 motifs distant from the TSS, PPFP tends to act in a repressive manner. Furthermore, 11 of these 16 gene sets contain the words *morphogenesis, development* or *organ formation* (Table 5A), implying that the effects of PAX8 binding at a distance >10kb upstream from TSS's are primarily anti-differentiation effects of PPFP.

**Table 5A:** Gene sets enriched in PPFP peaks with PAX8 motifs >10kb upstream from TSS's and differentially expressed in PPFP cells versus EV cells without pioglitazone

Gene set ID	Description	*q*-value ChIP	*q*-value RNA-seq PPFP *vs* EV without pioglitazone	Status PPFP *vs* EV
GO:0048598	embryonic morphogenesis	0.017	0.0014	repressed
GO:0022603	regulation of anatomical structure morphogenesis	0.013	0.0019	repressed
GO:0003007	heart morphogenesis	0.040	0.0023	repressed
GO:0072358	cardiovascular system development	0.029	0.0036	repressed
GO:0035239	tube morphogenesis	0.019	0.0037	repressed
GO:0060562	epithelial tube morphogenesis	0.046	0.0049	repressed
GO:0031330	negative regulation of cellular catabolic process	0.029	0.010	repressed
GO:0007507	heart development	0.016	0.013	repressed
GO:0035295	tube development	0.0090	0.014	repressed
GO:0010463	mesenchymal cell proliferation	0.0041	0.017	repressed
GO:0048645	organ formation	0.025	0.019	repressed
GO:0061061	muscle structure development	0.037	0.022	repressed
GO:0051240	positive regulation of multicellular organismal process	0.038	0.023	repressed
GO:0009895	negative regulation of catabolic process	0.041	0.028	repressed
GO:0010464	regulation of mesenchymal cell proliferation	0.016	0.031	repressed
GO:0009887	organ morphogenesis	0.012	0.035	repressed

All 16 gene sets are repressed by PPFP.

In contrast, analysis of PPFP peaks with PAX8 motifs ≤10kb from TSS's yielded 4 gene sets that were enriched in the ChIP and induced by PPFP, and no gene sets that were enriched and repressed. Thus, at the gene set level, there is a complete separation of PPFP as a repressor when the target genes have peaks with PAX8 motifs >10kb upstream from the TSS, versus an activator when the PAX8 peaks are ≤10kb. The 4 induced gene sets all relate to mitochondria and lipids (Table 5B), and contain 24 unique genes. In contrast to the analysis of PPFP peaks with PAX8 motifs, analysis of peaks with PPARG motifs did not identify differences in gene set activation versus repression based on distance from the TSS (data not shown).

**Table 5B:** Gene sets enriched in PPFP peaks with PAX8 motifs ≤10 kb from TSS's and differentially expressed in PPFP cells versus EV cells without pioglitazone

Gene set ID	Description	*q*-value ChIP	*q*-value RNA-seq PPFP *vs* EV without pioglitazone	Status PPFP *vs* EV
GO:0005740	mitochondrial envelope	0.047	6.49E-04	induced
GO:0044429	mitochondrial part	0.033	7.19E-04	induced
GO:0031966	mitochondrial membrane	0.047	8.51E-04	induced
GO:0005811	lipid particle	0.011	8.87E-04	induced

All 4 gene sets are induced by PPFP.

### Why is pioglitazone adipogenic in PPFP-expressing cells?

When mice with PPFP thyroid carcinomas are treated with pioglitazone, metastatic disease is prevented and the primary thyroid tumors shrink markedly [11]. The most striking part of the response is that pioglitazone is highly adipogenic, causing large accumulations of intracellular lipid and the induction of numerous adipocyte PPARG target genes in the thyroids. In contrast, pioglitazone has no effect on the thyroid glands of control mice. The induction of adipocyte genes is a hallmark of the pioglitazone response in cultured PPFP cells as shown here, too (Tables 3 and S2). Since PPARG is the master regulator of adipogenesis, the data indicate that pioglitazone turns PPFP into a strongly PPARG-like transcription factor. However, PPARG also is expressed in macrophages, where it plays an important role in promoting an anti-inflammatory “M2” phenotype [10]. Why does PPFP favor the induction of an adipocyte phenotype over a macrophage phenotype in the thyroid? To begin to understand this, we took advantage of the fact that a PPARG ChIP-seq analysis has been published comparing a mouse adipocyte cell line with mouse macrophages [29]. This study thus identified genes with PPARG peaks in mouse adipocytes but not macrophages, and vice versa. Using HomoloGene, we assessed the overlap between mouse genes with a nearby (≤10kb from TSS) PPARG peak and rat genes with at least one nearby PPFP peak. We found that PPFP binds near 34% of homologs with an adipocyte PPARG peak versus only 25% of homologs with a macrophage PPARG peak (Supplemental Table S4, *p* = 0.0022). The fact that PPFP in the thyroid preferentially binds to adipocyte PPARG target genes likely underlies the observation that the pioglitazone response is adipocyte-like.

### Why is pioglitazone therapeutic in the mouse model of PPFP thyroid carcinoma?

We reasoned that genes or pathways regulated in opposite directions by PPFP without pioglitazone versus PPFP with pioglitazone may be involved in the therapeutic efficacy of this drug. We therefore identified GO or KEGG terms in our RNA-seq data that are both induced (or repressed) in PPFP cells versus EV cells without pioglitazone, and repressed (or induced) in PPFP cells with pioglitazone versus PPFP cells without pioglitazone. Only three gene sets qualified, and all were induced by PPFP without pioglitazone and repressed by pioglitazone. The 3 gene sets relate to oxidative stress: *Glutathione metabolism* (KEGG), *peroxidase activity* (GO) and *arachidonic acid metabolism* (KEGG). There are 49 unique, differentially expressed genes within these gene sets, including multiple glutathione peroxidases, glutathione reductase, glutathione synthase, glutathione S-transferases, and peroxiredoxins (Supplemental Table S5). Based on these data, we hypothesized that PPFP induces oxidative stress and that pioglitazone impairs the ability of the cell to mount an appropriate antioxidant response.

**Figure 6:**
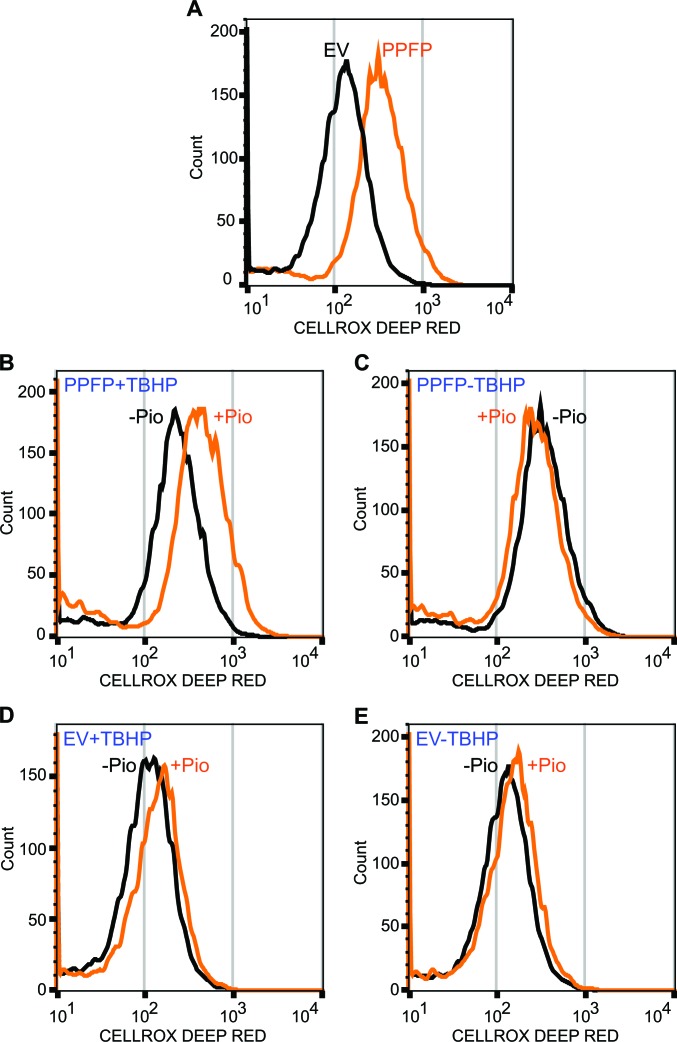
Analysis of oxidative stress in PPFP and EV cells The cells were incubated with the reactive oxygen species-sensitive dye CellROX Deep Red and analyzed by flow cytometry. **A.** PPFP cells have increase ROS relative to EV cells (cultured without pioglitazone). **B.** Pioglitazone (pio) increases ROS in PPFP cells cultured with tert-butyl hydroperoxide (TBHP). **C.** Pioglitazone does not increase ROS in PPFP cells cultured without TBHP. **D**, **E.** Pioglitazone does not increase ROS in EV cells. All cells (A-E) were cultured and analyzed at the same time. The EV cell tracing in A is the same preparation of cells as shown in E without pioglitazone, and the PPFP cell tracing in A is the same as that in C without pioglitazone.

To assess this, we evaluated oxidative stress in PPFP and EV cells by flow cytometry after treatment with the reactive oxygen species (ROS)-sensitive dye CellROX Deep Red. PPFP cells had greater ROS than EV cells, cultured in the absence of pioglitazone (Figure 6A). Analysis of PPFP cells cultured ± a low dose (50 μM) of the reactive peroxide tert-butyl hydroperoxide (TBHP) and pioglitazone showed that pioglitazone increased ROS in the presence of TBHP (Figure 6B) but not in its absence (Figure 6C). In contrast, in EV cells, pioglitazone did not increase ROS regardless of the presence or absence of TBHP (Figure 6D, 6E). These data support the hypothesis that PPFP induces oxidative stress, and that pioglitazone increases the susceptibility of PPFP cells to further oxidative stress. The data suggest that the therapeutic efficacy of pioglitazone in PPFP thyroid cancer may at least in part relate to synergism with cellular stressors to induce oxidative stress, cytotoxicity and ultimately, cell death.

### Similarity of gene regulation by PPFP in PCCL3 cells and human thyroid carcinomas

To judge the clinical relevance of the PPFP cell ChIP-seq and RNA-seq analyses, it would be ideal to compare these data to similar data from human PPFP carcinomas. However, human ChIP-seq data would be difficult if not impossible to obtain due to the fact that antibodies to endogenous PPFP also bind to PAX8 or PPARG (our PPFP is epitope-tagged), as well as the fact that PPFP thyroid carcinomas are uncommon. Furthermore, the largest gene expression profiling study of human PPFP thyroid carcinomas included only 7 cases [5]. This study identified 275 genes that were differentially expressed in 7 PPFP follicular carcinomas versus 82 non-PPFP thyroid tumors and 4 normal thyroids. Of those 275 genes, 264 have rat homologs, and we found a 31% overlap with differential expression in our RNA-seq data and 22% overlap with our ChIP-seq data (Supplemental Table S6). This overlap includes numerous PPARG target genes, including *ANGPTL4* and *AQP7*, which were two of the six most highly induced genes in the human PPFP carcinomas. We also found that the set of 49 ROS-related genes described above is induced in the 7 PPFP follicular carcinomas versus the non-PPFP follicular carcinomas (*p* = 0.0007, Fisher's exact test, Supplemental Table S7), suggesting that PPFP also causes increased ROS in human thyroid carcinomas.

## DISCUSSION

PPFP is an unusual oncoprotein in that it is the fusion of two transcription factors, PAX8 and PPARG, and it retains the DBDs of both parent proteins. However, until now there were no studies to determine whether PPFP actively bound both PAX8 and PPARG target genes. Gene expression data in PPFP thyroid carcinomas also are very limited - the largest study included only 7 PPFP carcinomas [5], and given patient heterogeneity, only limited conclusions can be drawn.

Here, we identified ~20,000 putative PPFP binding sites in the rat PCCL3 cell line genome, and found that these binding sites are enriched within −5 kb of transcription start sites and in first introns. PPFP peaks encompass known PAX8 and PPARG binding sites, indicating that both DBDs within PPFP are functional. Interestingly, PPFP preferentially binds to the subset of PAX8 peaks that also contain PPARG motifs, implying that the PPARG portion of PPFP is particularly important in directing PPFP to its target genes. The fact that many PAX8 binding sites have a nearby PPARG motif also suggests PPARG may play a role in normal thyroid biology. Thyroid-specific deletion of murine *Pparg* has not been reported, but would provide a means to address this question.

RNA-seq analysis shows that PPFP regulates the expression of ~1500 genes in the absence of pioglitazone and ~2000 genes in its presence. In general, pioglitazone reinforces PPFP-dependent gene expression, but in 17% of cases it reverses the effects of PPFP. PPFP regulates many genes known to be regulated by PAX8 in thyrocytes or PPARG in adipocytes. For the latter, the gene regulation by PPFP is particularly striking in the presence of pioglitazone. Although gene expression data in human PPFP thyroid carcinomas are very limited, there is excellent overlap between the human data and our data, including the induction of PPARG target genes.

We identified an unusual dichotomy in the function of PPFP for genes with peaks containing PAX8 motifs. When such motifs are located >10kb upstream from the TSS, the functional consequence tends to be gene set repression, including processes related to morphogenesis and development. In contrast, when such peaks are close to the TSS, the consequence tends to be gene set induction. This dichotomy likely reflects the summed activity of transcriptional activators and repressors brought to the target gene, but the factors that determine these differences are unknown.

The data are relevant to the biology of PPFP in human thyroid cancer. For example, PPFP induces gene sets related to the cell cycle, and represses gene sets related to differentiation. In a transgenic mouse model of PPFP thyroid carcinoma, pioglitazone was highly therapeutic [11], and this has led to a clinical trial in patients (clinicaltrials.gov identifier NCT01655719). A remarkable aspect of this response is that the drug trans-differentiated the surviving thyroid cancer cells into adipocyte-like cells. Our ChIP-seq data show that, in PCCL3 thyroid cells, PPFP binds to adipocyte PPARG target genes in preference to macrophage PPARG target genes, likely explaining why pioglitazone is specifically pro-adipogenic.

It is plausible that the therapeutic efficacy of pioglitazone is at least in part due to its adipogenic pro-differentiation effects. However, our data uncovered another potential contributing factor. We found that PPFP cells have higher expression of ROS-related genes than EV cells, and this reflects higher levels of oxidative stress. Pioglitazone caused the PPFP cells to develop even greater levels of ROS when exposed to a low dose of the reactive peroxide TBHP, indicating that pioglitazone sensitizes PPFP cells to potential oxidant stressors. These data suggest pioglitazone might sensitize PPFP thyroid cancers *in vivo* to oxidative stressors, leading to increased cytotoxicity and cell death. Substantiation of this hypothesis could lead to approaches to further enhance the efficacy of this drug.

## MATERIALS AND METHODS

### Cell culture

PCCL3-PPFP cells stably express human PPFP with a 3xMyc tag at the amino terminus, and PCCL3-EV cells have been stably transfected with the empty vector [12]. PPFP and EV cells were cultured as previously described [12]. In some experiments, the cells were treated with 1 μM pioglitazone (from a 1 mM stock solution in DMSO) or vehicle for the times indicated prior to harvest.

### Antibodies

Antibodies were obtained from the following sources as indicated: Cell Signaling Technology (Danvers, MA) - beta actin #8457, Ccnb1 #12231, Hes1 #11988, Myc tag #2276, Notch1 #3608 and Plk3 #4896; Proteintech (Chicago, IL) - Acaa2 #11111-1-AP and Icam5 #12759-1-AP; Sigma (St. Louis, MO) - Foxe1 #SAB2100840; and Life Technologies (Grand Island, NY) - Cdk1 #MA5-11472 and Plin1 #PA1-1051.

### Flow cytometry

DNA content was analyzed by propidium iodide staining and oxidative stress was analyzed with CellROX Deep Red per the vendor's protocols (Life Technologies). For the oxidative stress experiments, the cells were cultured ±1 μM pioglitazone for 2 days and ±50 μM TBHP for the final hour. Approximately 10,000 cells per condition were analyzed in the University of Michigan Comprehensive Cancer Center Flow Cytometry Core on a MACSQuant cytometer.

### ChIP-seq assay

PPFP cells were cultured with pioglitazone for 16 hours, crosslinked with formaldehyde, sonicated to an average DNA size of 300 to 500 bp, and immunoprecipitated with anti-Myc antibody at 1:500 overnight at 4C using the protocol of Upstate Biotechnology (Lake Placid, NY), except that immunoprecipitation was performed with Dynabeads G (Life Technologies). ChIP and input DNA were used for next generation library construction and DNA sequencing on an Illumina HiSeq 2000 per the manufacturer's protocol using 50 nt single-end reads, performed by the University of Michigan DNA Sequencing Core. Four samples were barcoded and run on one lane, obtaining an average of 31 million reads per sample.

### RNA-seq assay

PPFP and EV cells were treated with 1 μM pioglitazone or vehicle for 16 hours. Total RNA was prepared using an RNeasy Mini Kit (Qiagen). Three independent experiments were performed. Library construction (Illumina TruSeq RNA) and sequencing on an Illumina HiSeq2000 using 50 nt paired end reads were per the manufacturer's protocols, performed by the University of Michigan DNA Sequencing Core. The samples were barcoded and loaded onto the same run, with all samples from each experiment run on the same two lanes; an average of 88 million reads were obtained per sample.

PPFP ChIP-seq and RNA-seq data were deposited in Gene Expression Omnibus (GEO) with the accession ID GSE70354.

### ChIP-seq data analysis

The quality of reads were assessed using FastQC [30]. There were 29.3 and 33.5 million reads sequenced for PPFP ChIP and input samples, respectively. ChIP-seq and input reads were aligned to the rat reference genome (rn4) using BWA (version 0.5.9-r16) with default options. MACS2 (2.0.10.20131216beta) was used to call peaks using a *q*-value < 0.05 cutoff [31]. Peaks were then filtered by PePr (1.0.5) [32] to remove artifacts due to high PCR duplications. Peak boundaries were re-defined as 150 bp from the peak mode, and over-represented motifs were identified from the peaks by MEME(4.9.1), searching for the top 10 motifs with minimal width of 10bp and maximal width of 18bp. The most over-represented motif (shown in Figure 5A) was very close to the PPARG motif previously reported [24, 29, 33] and was used as the position weight matrix (PWM) for the PPARG motif in all following analyses. Motif occurrences in the peaks were detected by FIMO(4.9.1) using default parameters and the PWM output from MEME. The presence of PPARG and PAX8 motifs in PPFP peaks was detected with the MEME/FIMO suite, and was also independently discovered using another motif discovery tool, HOMER [25], as the top 2 known enriched motifs. Of the top 35 motifs found by HOMER, none were related to Myc, assuring that potential nonspecific binding caused by using a Myc tag antibody should be negligible.

Peaks were annotated to the genome with respect to gene features using an adapted HOMER script. If a peak had two or more annotations, a priority was assigned based on the order from left to right in Figure 4, as follows: −1 to +1kb (relative to the TSS), −1 to −5kb, −5 to −10kb, exon, UTR, intron, and intergenic. We define “intergenic” as outside of the region between 10kb upstream from a TSS and its 3′UTR.

PAX8 ChIP-seq raw read data (GSE26871) were downloaded and analyzed as described above. The PAX8 motif (shown in Figure 5A) was found as the top hit by MEME searching for the top 10 overrepresented motifs with minimal width of 10bp and maximal width of 15bp. The PAX8 motif identified here closely matches that previously published [23].

PPARG ChIP-seq peaks from mouse adipocyte and macrophage cells were downloaded from GEO (GSE21314).

### RNA-seq data analysis

Quality checks were performed on RNA-seq reads with RSeQC (2.3.9) [34]. The reads were aligned to rn4 with tophat2 (v2.0.11) and gene read counts were quantified by HTseq (0.6.1p1) [35] with option “-m intersection-strict” and normalized using the *edgeR* (3.2.4) Bioconductor package [36]. Differential expression analysis was performed using edgeR with tagwise dispersion for each pairwise comparison of PPFP cells or EV cells treated with and without pioglitazone (four comparisons total). False discovery rate (FDR) was controlled using the Benjamini-Hochberg method [37].

### Gene set enrichment testing

*P*-values from differential expression analysis from *edgeR* using the RNA-seq data were input into LRpath [16, 17] for gene set enrichment testing. LRpath is a logistic-regression-based method that models the relationship between the log-odds of genes belonging to a gene set and their -log(*p*-values). We used the directional test option in LRpath, and tested GO terms and KEGG pathways with each pairwise comparison of PPFP cells or EV cells treated with and without pioglitazone. Gene sets satisfying FDR≤0.05 were considered to be significant.

Gene set enrichment testing of the ChIP-seq data was performed with ChIP-Enrich, a logistic-regression-based method that uses a smoothing spline to empirically adjust for gene locus length and mappability [28]. Gene set enrichment results were corrected for multiple testing using the Benjamini-Hochberg FDR correction. Only gene sets with ≤500 genes were reported, as gene sets with larger numbers of genes are more general and provide limited biological insight.

## SUPPLEMENTARY MATERIAL TABLES


