# Supplementary material for: Genomic binding and regulation of gene expression by the thyroid carcinoma-associated PAX8-PPARG fusion protein
Source: Oncotarget. 2015 Nov 16;6(38):40418–32. doi: 10.18632/oncotarget.6340 (PMC4747342; doi:10.18632/oncotarget.6340)
Supplement: Supplementary file 1 [file oncotarget-06-40418-s001.pdf]

Genomic binding and regulation of gene expression by the thyroid carcinoma-associated PAX8-PPARG fusion protein

Supplementary Material

Table S1. Gene sets significantly enriched in PPFP cells versus EV cells without pioglitazone

Concept.ID	Concept.name	p-value	q-value	Status PPFP vs EV
GO:0071346	cellular response to interferon-gamma	6.17E-07	1.37E-04	up
GO:0030529	ribonucleoprotein complex	8.09E-06	1.71E-04	up
GO:0005740	mitochondrial envelope	5.07E-05	6.49E-04	up
GO:0005811	lipid particle	8.27E-05	8.87E-04	up
GO:0046689	response to mercury ion	1.72E-05	1.05E-03	up
GO:0005730	nucleolus	2.08E-04	2.04E-03	up
GO:0004364	glutathione transferase activity	3.24E-05	2.25E-03	up
rno00480	Glutathione metabolism	1.29E-05	2.48E-03	up
GO:0042555	MCM complex	2.92E-04	2.54E-03	up
GO:0071219	cellular response to molecule of bacterial origin	6.99E-05	2.75E-03	up
GO:0071398	cellular response to fatty acid	1.02E-04	3.35E-03	up
GO:0006396	RNA processing	1.22E-04	3.56E-03	up
GO:0071384	cellular response to corticosteroid stimulus	1.23E-04	3.56E-03	up
GO:0000502	proteasome complex	5.06E-04	3.95E-03	up
GO:0006457	protein folding	2.13E-04	5.08E-03	up
GO:0005504	fatty acid binding	1.50E-04	5.22E-03	up
GO:0004601	peroxidase activity	1.68E-04	5.30E-03	up
GO:0022613	ribonucleoprotein complex biogenesis	3.45E-04	6.94E-03	up
GO:0010226	response to lithium ion	4.33E-04	7.95E-03	up
GO:0070585	protein localization to mitochondrion	4.58E-04	8.24E-03	up
GO:0051082	unfolded protein binding	4.02E-04	9.01E-03	up
GO:0031072	heat shock protein binding	4.63E-04	9.65E-03	up
GO:0016817	hydrolase activity, acting on acid anhydrides	4.93E-04	9.65E-03	up
GO:0019915	lipid storage	6.11E-04	1.03E-02	up
GO:0002673	regulation of acute inflammatory response	6.27E-04	1.04E-02	up
GO:0071347	cellular response to interleukin-1	7.00E-04	1.13E-02	up
GO:0007338	single fertilization	7.94E-04	1.23E-02	up
GO:0009263	deoxyribonucleotide biosynthetic process	8.57E-04	1.31E-02	up
GO:0006260	DNA replication	1.16E-03	1.62E-02	up
GO:0001659	temperature homeostasis	1.28E-03	1.71E-02	up
GO:0031577	spindle checkpoint	1.33E-03	1.73E-02	up
rno00590	Arachidonic acid metabolism	3.63E-04	1.75E-02	up
GO:0043604	amide biosynthetic process	1.66E-03	2.01E-02	up
GO:0031000	response to caffeine	2.15E-03	2.33E-02	up
GO:0007059	chromosome segregation	2.16E-03	2.34E-02	up
GO:0046683	response to organophosphorus	2.52E-03	2.54E-02	up
GO:0016071	mRNA metabolic process	2.60E-03	2.60E-02	up
GO:0019400	alditol metabolic process	2.74E-03	2.71E-02	up
GO:0006730	one-carbon metabolic process	2.87E-03	2.78E-02	up
GO:0000278	mitotic cell cycle	3.67E-03	3.31E-02	up
GO:0071354	cellular response to interleukin-6	3.89E-03	3.43E-02	up
GO:0043295	glutathione binding	4.54E-03	4.00E-02	up
GO:0044265	cellular macromolecule catabolic process	4.97E-03	4.10E-02	up
GO:0006220	pyrimidine nucleotide metabolic process	5.00E-03	4.11E-02	up

GO:0035173	histone kinase activity	5.04E-03	4.32E-02	up
GO:0002020	protease binding	5.15E-03	4.36E-02	up
GO:0043236	laminin binding	5.42E-03	4.43E-02	up
GO:0030163	protein catabolic process	5.61E-03	4.46E-02	up
GO:0000187	activation of MAPK activity	5.80E-03	4.57E-02	up
GO:0031625	ubiquitin protein ligase binding	5.88E-03	4.64E-02	up
GO:0004298	threonine-type endopeptidase activity	6.16E-03	4.67E-02	up
GO:0004867	serine-type endopeptidase inhibitor activity	6.67E-03	4.77E-02	up
GO:0004091	carboxylesterase activity	6.73E-03	4.77E-02	up
GO:0000775	chromosome, centromeric region	8.42E-03	4.79E-02	up
rno03050	Proteasome	1.44E-03	4.81E-02	up
GO:0008021	synaptic vesicle	1.36E-14	6.25E-12	down
GO:0007267	cell-cell signaling	1.98E-09	7.03E-06	down
GO:0004888	transmembrane signaling receptor activity	3.96E-08	2.75E-05	down
GO:0048858	cell projection morphogenesis	3.74E-08	2.95E-05	down
GO:0048730	epidermis morphogenesis	4.75E-08	2.95E-05	down
GO:0044306	neuron projection terminus	1.12E-06	3.45E-05	down
GO:0050808	synapse organization	1.10E-07	4.87E-05	down
GO:0048667	cell morphogenesis involved in neuron differentiation	1.38E-07	4.92E-05	down
GO:0004872	receptor activity	1.56E-07	5.40E-05	down
GO:0043534	blood vessel endothelial cell migration	2.87E-07	8.80E-05	down
GO:1901342	regulation of vasculature development	5.06E-07	1.28E-04	down
GO:0045995	regulation of embryonic development	1.15E-06	1.77E-04	down
GO:0090288	negative regulation of cellular response to growth factor stimulus	2.02E-06	2.55E-04	down
GO:0007167	enzyme linked receptor protein signaling pathway	2.91E-06	3.13E-04	down
GO:0051960	regulation of nervous system development	3.10E-06	3.24E-04	down
GO:0010507	negative regulation of autophagy	4.55E-06	4.25E-04	down
GO:0008328	ionotropic glutamate receptor complex	2.62E-05	4.47E-04	down
GO:0046873	metal ion transmembrane transporter activity	2.88E-06	6.67E-04	down
GO:0072001	renal system development	9.32E-06	7.18E-04	down
GO:0044448	cell cortex part	6.52E-05	7.51E-04	down
GO:0005262	calcium channel activity	6.92E-06	1.20E-03	down
GO:0005179	hormone activity	9.72E-06	1.35E-03	down
GO:0048598	embryonic morphogenesis	2.40E-05	1.39E-03	down
GO:0005216	ion channel activity	2.59E-05	2.00E-03	down
GO:0072511	divalent inorganic cation transport	4.27E-05	2.12E-03	down
GO:0030001	metal ion transport	4.56E-05	2.12E-03	down
GO:0030031	cell projection assembly	5.53E-05	2.42E-03	down
GO:0031344	regulation of cell projection organization	5.95E-05	2.57E-03	down
GO:0007033	vacuole organization	7.69E-05	2.87E-03	down
GO:0007224	smoothened signaling pathway	8.22E-05	2.97E-03	down
GO:0005773	vacuole	4.12E-04	3.33E-03	down
GO:0021675	nerve development	9.94E-05	3.35E-03	down
GO:0060589	nucleoside-triphosphatase regulator activity	8.58E-05	3.54E-03	down
GO:0033280	response to vitamin D	1.21E-04	3.56E-03	down
GO:1900449	regulation of glutamate receptor signaling pathway	1.31E-04	3.69E-03	down
GO:0048736	appendage development	1.37E-04	3.75E-03	down
GO:0002067	glandular epithelial cell differentiation	1.39E-04	3.75E-03	down
GO:0007517	muscle organ development	1.40E-04	3.75E-03	down
GO:0045838	positive regulation of membrane potential	1.64E-04	4.25E-03	down
rno04514	Cell adhesion molecules (CAMs)	4.55E-05	4.39E-03	down
GO:0007155	cell adhesion	1.74E-04	4.44E-03	down
GO:0001763	morphogenesis of a branching structure	2.40E-04	5.45E-03	down
GO:0048483	autonomic nervous system development	2.86E-04	6.22E-03	down
GO:0005246	calcium channel regulator activity	2.83E-04	6.54E-03	down
GO:0010714	positive regulation of collagen metabolic process	3.18E-04	6.60E-03	down

GO:0050886	endocrine process	3.48E-04	6.94E-03	down
GO:0005891	voltage-gated calcium channel complex	1.04E-03	7.77E-03	down
GO:0030175	filopodium	1.06E-03	7.79E-03	down
GO:0060359	response to ammonium ion	4.33E-04	7.95E-03	down
GO:0031012	extracellular matrix	1.12E-03	8.07E-03	down
GO:0021510	spinal cord development	4.86E-04	8.69E-03	down
GO:0034405	response to fluid shear stress	5.63E-04	9.68E-03	down
GO:0070372	regulation of ERK1 and ERK2 cascade	5.86E-04	1.00E-02	down
GO:0005158	insulin receptor binding	6.49E-04	1.15E-02	down
GO:0030029	actin filament-based process	7.26E-04	1.16E-02	down
GO:0004714	transmembrane receptor protein tyrosine kinase activity	7.88E-04	1.27E-02	down
rno04340	Hedgehog signaling pathway	2.13E-04	1.37E-02	down
GO:0060021	palate development	9.84E-04	1.42E-02	down
GO:0031418	L-ascorbic acid binding	9.75E-04	1.54E-02	down
GO:0005089	Rho guanyl-nucleotide exchange factor activity	1.02E-03	1.58E-02	down
GO:0019838	growth factor binding	1.07E-03	1.61E-02	down
GO:0048863	stem cell differentiation	1.17E-03	1.62E-02	down
GO:0044087	regulation of cellular component biogenesis	1.17E-03	1.62E-02	down
GO:0048286	lung alveolus development	1.31E-03	1.71E-02	down
GO:0009100	glycoprotein metabolic process	1.31E-03	1.71E-02	down
GO:0048259	regulation of receptor-mediated endocytosis	1.37E-03	1.75E-02	down
GO:0015175	neutral amino acid transmembrane transporter activity	1.25E-03	1.81E-02	down
GO:0019430	removal of superoxide radicals	1.43E-03	1.81E-02	down
GO:0043618	regulation of transcription from RNA polymerase II promoter in response to stress	1.48E-03	1.87E-02	down
GO:0035567	non-canonical Wnt receptor signaling pathway	1.50E-03	1.88E-02	down
GO:0031623	receptor internalization	1.52E-03	1.90E-02	down
GO:0005044	scavenger receptor activity	1.36E-03	1.91E-02	down
GO:0006109	regulation of carbohydrate metabolic process	1.57E-03	1.93E-02	down
GO:0035023	regulation of Rho protein signal transduction	1.66E-03	2.01E-02	down
GO:0008201	heparin binding	1.57E-03	2.06E-02	down
GO:0052866	phosphatidylinositol phosphate phosphatase activity	1.66E-03	2.10E-02	down
GO:0043462	regulation of ATPase activity	1.81E-03	2.11E-02	down
GO:0048732	gland development	1.86E-03	2.13E-02	down
GO:0051248	negative regulation of protein metabolic process	1.87E-03	2.13E-02	down
GO:0048771	tissue remodeling	1.99E-03	2.23E-02	down
GO:0048538	thymus development	2.00E-03	2.23E-02	down
GO:2000241	regulation of reproductive process	2.00E-03	2.23E-02	down
GO:0072503	cellular divalent inorganic cation homeostasis	2.02E-03	2.23E-02	down
GO:0021904	dorsal/ventral neural tube patterning	2.23E-03	2.37E-02	down
GO:0007218	neuropeptide signaling pathway	2.23E-03	2.37E-02	down
GO:0048546	digestive tract morphogenesis	2.25E-03	2.38E-02	down
GO:0010517	regulation of phospholipase activity	2.36E-03	2.46E-02	down
GO:0072091	regulation of stem cell proliferation	2.40E-03	2.49E-02	down
GO:0050919	negative chemotaxis	2.54E-03	2.55E-02	down
GO:0016878	acid-thiol ligase activity	2.56E-03	2.91E-02	down
GO:0052745	inositol phosphate phosphatase activity	2.80E-03	2.99E-02	down
GO:0001649	osteoblast differentiation	3.37E-03	3.10E-02	down
GO:2000179	positive regulation of neural precursor cell proliferation	3.52E-03	3.21E-02	down
GO:0014072	response to isoquinoline alkaloid	3.73E-03	3.35E-02	down
GO:2000378	negative regulation of reactive oxygen species metabolic process	3.93E-03	3.46E-02	down
GO:0001158	enhancer sequence-specific DNA binding	3.64E-03	3.51E-02	down
GO:0050867	positive regulation of cell activation	4.06E-03	3.54E-02	down
GO:0048663	neuron fate commitment	4.08E-03	3.54E-02	down
GO:0015296	anion:cation symporter activity	4.04E-03	3.69E-02	down
GO:0033673	negative regulation of kinase activity	4.79E-03	4.02E-02	down

GO:0034698	response to gonadotropin stimulus	5.10E-03	4.19E-02	down
GO:0070528	protein kinase C signaling cascade	5.42E-03	4.36E-02	down
GO:0004559	alpha-mannosidase activity	5.36E-03	4.43E-02	down
GO:0035254	glutamate receptor binding	6.18E-03	4.67E-02	down
GO:0005507	copper ion binding	6.78E-03	4.77E-02	down
GO:0000982	RNA polymerase II core promoter proximal region sequence-specific DNA binding transcription factor activity	7.00E-03	4.77E-02	down
rno00511	Other glycan degradation	1.50E-03	4.81E-02	down

Table S2. Gene sets enriched in the comparison of PPFP cells cultured with versus without pioglitazone

**Concept.ID**	**Concept.name**	**p.value_PPFP_pio_vs_PPFP_nopio**	**FDR_PPFP_pio_vs_PPFP_nopio**	**status_PPFP_pio_vs_PPFP_nopio**
GO:0009062	fatty acid catabolic process	7.90E-14	7.00E-11	up
GO:0019395	fatty acid oxidation	3.87E-13	1.53E-10	up
GO:0004091	carboxylesterase activity	2.77E-12	1.93E-09	up
rno03320	PPAR signaling pathway	2.36E-10	4.55E-08	up
GO:0006637	acyl-CoA metabolic process	1.15E-09	2.15E-07	up
GO:0006641	triglyceride metabolic process	3.12E-09	4.81E-07	up
GO:0005777	peroxisome	4.87E-09	5.61E-07	up
GO:0005740	mitochondrial envelope	7.76E-09	5.96E-07	up
GO:0005759	mitochondrial matrix	1.56E-08	1.03E-06	up
GO:0071398	cellular response to fatty acid	2.09E-08	2.74E-06	up
GO:0006577	amino-acid betaine metabolic process	5.36E-08	6.13E-06	up
GO:0005811	lipid particle	4.64E-07	1.94E-05	up
GO:0007494	midgut development	3.03E-07	2.86E-05	up
GO:0003995	acyl-CoA dehydrogenase activity	1.24E-07	2.87E-05	up
GO:0008374	O-acyltransferase activity	1.90E-07	3.30E-05	up
GO:0033555	multicellular organismal response to stress	1.62E-06	0.0001223	up
GO:0047617	acyl-CoA hydrolase activity	1.57E-06	0.0001559	up
GO:0033764	steroid dehydrogenase activity, acting on the CH-OH group of donors, NAD or NADP as acceptor	1.85E-06	0.0001602	up
rno00071	Fatty acid metabolism	4.71E-06	0.0002273	up
rno00561	Glycerolipid metabolism	1.07E-05	0.0002965	up
rno04146	Peroxisome	1.08E-05	0.0002965	up
GO:0050662	coenzyme binding	5.28E-06	0.0003056	up
rno01040	Biosynthesis of unsaturated fatty acids	2.47E-05	0.0004107	up
GO:0046364	monosaccharide biosynthetic process	1.42E-05	0.0005668	up
rno00280	Valine, leucine and isoleucine degradation	5.67E-05	0.0007963	up
GO:0048806	genitalia development	3.61E-05	0.0011121	up
rno00830	Retinol metabolism	0.000160094	0.0020272	up
GO:0090181	regulation of cholesterol metabolic process	0.000140276	0.0034523	up
GO:0055088	lipid homeostasis	0.000148091	0.0035948	up
GO:0033762	response to glucagon stimulus	0.000167044	0.0039732	up
GO:0007031	peroxisome organization	0.000182955	0.0042657	up
GO:0042493	response to drug	0.00020957	0.0047917	up
rno00900	Terpenoid backbone biosynthesis	0.00113676	0.0087758	up
GO:0009950	dorsal/ventral axis specification	0.00045957	0.008949	up
GO:0052646	alditol phosphate metabolic process	0.000479803	0.0092919	up
GO:0032365	intracellular lipid transport	0.000490656	0.0094505	up
GO:2000811	negative regulation of anoikis	0.000659792	0.0116682	up
GO:0030176	integral to endoplasmic reticulum membrane	0.001305239	0.0122799	up
GO:0034383	low-density lipoprotein particle clearance	0.000890003	0.0145345	up
rno00564	Glycerophospholipid metabolism	0.002406565	0.0165881	up
GO:0055067	monovalent inorganic cation homeostasis	0.001192692	0.0182194	up
GO:0019915	lipid storage	0.00150537	0.0217756	up
rno00650	Butanoate metabolism	0.003825672	0.0254605	up
GO:0046683	response to organophosphorus	0.002255279	0.0292733	up
GO:0070849	response to epidermal growth factor stimulus	0.002534691	0.0315323	up
GO:0043277	apoptotic cell clearance	0.002793442	0.0342859	up
GO:0032846	positive regulation of homeostatic process	0.002828219	0.0345628	up
GO:0071384	cellular response to corticosteroid stimulus	0.002970917	0.0357146	up
GO:0050873	brown fat cell differentiation	0.002992652	0.0357146	up
GO:0051004	regulation of lipoprotein lipase activity	0.002995847	0.0357146	up
GO:2000351	regulation of endothelial cell apoptotic process	0.003525976	0.0397964	up

GO:0015850	organic hydroxy compound transport	0.003563181	0.0399618	up
GO:0031012	extracellular matrix	5.95E-08	2.74E-06	down
rno05140	Leishmaniasis	4.26E-08	4.11E-06	down
GO:0030029	actin filament-based process	2.25E-06	0.0001506	down
rno05323	Rheumatoid arthritis	3.41E-06	0.0002193	down
GO:0005201	extracellular matrix structural constituent	3.39E-06	0.0002355	down
rno00590	Arachidonic acid metabolism	6.77E-06	0.0002614	down
GO:0048667	cell morphogenesis involved in neuron differentiation	5.03E-06	0.000297	down
GO:0010714	positive regulation of collagen metabolic process	5.37E-06	0.000307	down
rno05145	Toxoplasmosis	1.48E-05	0.0003582	down
rno00480	Glutathione metabolism	2.32E-05	0.0004107	down
rno05150	Staphylococcus aureus infection	2.39E-05	0.0004107	down
rno05320	Autoimmune thyroid disease	2.55E-05	0.0004107	down
GO:0034405	response to fluid shear stress	9.89E-06	0.0004326	down
GO:0005509	calcium ion binding	9.59E-06	0.0004519	down
GO:0042611	MHC protein complex	4.29E-05	0.0007325	down
GO:0048858	cell projection morphogenesis	2.04E-05	0.0007529	down
rno04612	Antigen processing and presentation	5.78E-05	0.0007963	down
GO:0019882	antigen processing and presentation	3.39E-05	0.0010637	down
GO:0032940	secretion by cell	6.53E-05	0.0018357	down
rno05332	Graft-versus-host disease	0.000168061	0.0020272	down
GO:0005044	scavenger receptor activity	5.30E-05	0.0021656	down
GO:0031344	regulation of cell projection organization	8.21E-05	0.0022201	down
rno05330	Allograft rejection	0.000212994	0.0024181	down
GO:0010507	negative regulation of autophagy	0.000121092	0.0030677	down
GO:0051960	regulation of nervous system development	0.000136468	0.0034301	down
rno04060	Cytokine-cytokine receptor interaction	0.00032966	0.0035347	down
rno04672	Intestinal immune network for IgA production	0.000366505	0.0037229	down
GO:0007568	aging	0.000168872	0.0039899	down
rno05416	Viral myocarditis	0.000455994	0.0044003	down
rno04640	Hematopoietic cell lineage	0.000484261	0.0044506	down
rno04514	Cell adhesion molecules (CAMs)	0.000508445	0.0044605	down
GO:0001818	negative regulation of cytokine production	0.000234665	0.0052305	down
rno05144	Malaria	0.000679056	0.0056982	down
rno04940	Type I diabetes mellitus	0.000791432	0.0063644	down
GO:0051702	interaction with symbiont	0.00030831	0.0064713	down
GO:0044306	neuron projection terminus	0.000557189	0.0067596	down
GO:0017124	SH3 domain binding	0.000187394	0.0068448	down
GO:0009986	cell surface	0.000744638	0.008582	down
rno04145	Phagosome	0.001218685	0.0090464	down
GO:2000179	positive regulation of neural precursor cell proliferation	0.000539436	0.0101151	down
GO:0008201	heparin binding	0.000454226	0.0131347	down
rno00982	Drug metabolism - cytochrome P450	0.001970487	0.0140853	down
GO:0008081	phosphoric diester hydrolase activity	0.00055592	0.0154323	down
GO:0030199	collagen fibril organization	0.001256922	0.0190365	down
GO:0048286	lung alveolus development	0.001312782	0.0197979	down
GO:0051606	detection of stimulus	0.00134501	0.0199559	down
GO:0051015	actin filament binding	0.000808186	0.0215724	down
GO:0050776	regulation of immune response	0.0015564	0.0222415	down
GO:0048471	perinuclear region of cytoplasm	0.002566831	0.0227559	down
GO:0001975	response to amphetamine	0.001706658	0.0238126	down
GO:0060359	response to ammonium ion	0.001943929	0.026475	down
GO:0043425	bHLH transcription factor binding	0.001110572	0.0275263	down
GO:0048706	embryonic skeletal system development	0.002271492	0.0292733	down
GO:0042641	actomyosin	0.003922218	0.0301357	down
GO:0015103	inorganic anion transmembrane transporter	0.001595943	0.0335632	down

	activity			
GO:0048736	appendage development	0.003174979	0.0371357	down
GO:0001708	cell fate specification	0.003211873	0.0374437	down
GO:0031252	cell leading edge	0.005454577	0.0412223	down
GO:0021510	spinal cord development	0.004207237	0.0451042	down
rno00980	Metabolism of xenobiotics by cytochrome P450	0.007076013	0.0455224	down
GO:0015837	amine transport	0.004306295	0.0458304	down
GO:0042490	mechanoreceptor differentiation	0.004374863	0.0461444	down
GO:0002697	regulation of immune effector process	0.004653806	0.0487961	down
GO:0004601	peroxidase activity	0.002702283	0.0497887	down
GO:0003950	NAD+ ADP-ribosyltransferase activity	0.002726182	0.0497887	down

Table S3. GO terms enriched with PPFP peaks. Peaks were associated with the gene with the nearest transcription start site.

Geneset.ID	Description	q-value
GO:0030099	myeloid cell differentiation	2.84E-06
GO:0002520	immune system development	6.87E-06
GO:0008289	lipid binding	2.18E-05
GO:0030097	hemopoiesis	2.20E-05
GO:0048534	hemopoietic or lymphoid organ development	4.52E-05
GO:0030218	erythrocyte differentiation	7.08E-05
GO:0000122	negative regulation of transcription from RNA polymerase II promoter	2.18E-04
GO:0009968	negative regulation of signal transduction	3.08E-04
GO:0001932	regulation of protein phosphorylation	3.19E-04
GO:0034101	erythrocyte homeostasis	3.27E-04
GO:0032270	positive regulation of cellular protein metabolic process	3.45E-04
GO:0007167	enzyme linked receptor protein signaling pathway	3.50E-04
GO:0044255	cellular lipid metabolic process	3.52E-04
GO:0045637	regulation of myeloid cell differentiation	4.43E-04
GO:0032787	monocarboxylic acid metabolic process	5.18E-04
GO:0045597	positive regulation of cell differentiation	5.42E-04
GO:0016627	oxidoreductase activity, acting on the CH-CH group of donors	6.02E-04
GO:0031401	positive regulation of protein modification process	6.37E-04
GO:0010648	negative regulation of cell communication	6.59E-04
GO:0022603	regulation of anatomical structure morphogenesis	7.16E-04
GO:0005543	phospholipid binding	7.23E-04
GO:0044429	mitochondrial part	8.30E-04
GO:0055114	oxidation-reduction process	1.09E-03
GO:0050730	regulation of peptidyl-tyrosine phosphorylation	1.21E-03
GO:0010876	lipid localization	1.34E-03
GO:0042803	protein homodimerization activity	1.38E-03
GO:0023057	negative regulation of signaling	1.40E-03
GO:0002521	leukocyte differentiation	1.44E-03
GO:0019901	protein kinase binding	2.14E-03
GO:0016054	organic acid catabolic process	2.62E-03
GO:0019900	kinase binding	2.91E-03
GO:0002573	myeloid leukocyte differentiation	3.77E-03
GO:0044282	small molecule catabolic process	4.24E-03
GO:0043069	negative regulation of programmed cell death	4.63E-03
GO:0030334	regulation of cell migration	4.63E-03
GO:0050731	positive regulation of peptidyl-tyrosine phosphorylation	4.72E-03
GO:0043066	negative regulation of apoptotic process	4.79E-03
GO:0045639	positive regulation of myeloid cell differentiation	5.83E-03
GO:0006091	generation of precursor metabolites and energy	6.33E-03
GO:0001934	positive regulation of protein phosphorylation	6.45E-03
GO:0006631	fatty acid metabolic process	6.68E-03
GO:0060548	negative regulation of cell death	6.94E-03
GO:0045017	glycerolipid biosynthetic process	7.68E-03
GO:0051091	positive regulation of sequence-specific DNA binding transcription factor activity	7.70E-03
GO:0010608	posttranscriptional regulation of gene expression	7.93E-03
GO:0022900	electron transport chain	7.93E-03
GO:0018108	peptidyl-tyrosine phosphorylation	8.12E-03
GO:0000302	response to reactive oxygen species	8.37E-03
GO:0005811	lipid particle	8.77E-03
GO:0008610	lipid biosynthetic process	8.85E-03
GO:0022604	regulation of cell morphogenesis	8.85E-03
GO:0006869	lipid transport	8.88E-03
GO:0071495	cellular response to endogenous stimulus	8.93E-03
GO:0010627	regulation of intracellular protein kinase cascade	9.00E-03
GO:0006979	response to oxidative stress	1.00E-02
GO:0000082	G1/S transition of mitotic cell cycle	1.01E-02
GO:0071702	organic substance transport	1.01E-02
GO:2000145	regulation of cell motility	1.04E-02

GO:0051270	regulation of cellular component movement	1.04E-02
GO:0071345	cellular response to cytokine stimulus	1.07E-02
GO:0048872	homeostasis of number of cells	1.08E-02
GO:0007169	transmembrane receptor protein tyrosine kinase signaling pathway	1.09E-02
GO:0016055	Wnt receptor signaling pathway	1.09E-02
GO:0032870	cellular response to hormone stimulus	1.09E-02
GO:0061077	chaperone-mediated protein folding	1.12E-02
GO:0006520	cellular amino acid metabolic process	1.21E-02
GO:0019904	protein domain specific binding	1.28E-02
GO:0071453	cellular response to oxygen levels	1.30E-02
GO:0010562	positive regulation of phosphorus metabolic process	1.31E-02
GO:0051090	regulation of sequence-specific DNA binding transcription factor activity	1.31E-02
GO:0034330	cell junction organization	1.32E-02
GO:0045216	cell-cell junction organization	1.34E-02
GO:0006635	fatty acid beta-oxidation	1.35E-02
GO:0001667	ameboidal cell migration	1.37E-02
GO:0002761	regulation of myeloid leukocyte differentiation	1.46E-02
GO:0042327	positive regulation of phosphorylation	1.47E-02
GO:0034614	cellular response to reactive oxygen species	1.62E-02
GO:0008134	transcription factor binding	1.72E-02
GO:0032403	protein complex binding	1.73E-02
GO:0005912	adherens junction	1.82E-02
GO:0051015	actin filament binding	1.84E-02
GO:0004721	phosphoprotein phosphatase activity	1.84E-02
GO:0008144	drug binding	1.84E-02
GO:0016323	basolateral plasma membrane	1.84E-02
GO:0042493	response to drug	1.85E-02
GO:0072329	monocarboxylic acid catabolic process	1.87E-02
GO:0051085	chaperone mediated protein folding requiring cofactor	1.89E-02
GO:0001775	cell activation	1.92E-02
GO:0043549	regulation of kinase activity	1.97E-02
GO:0044242	cellular lipid catabolic process	2.05E-02
GO:0002696	positive regulation of leukocyte activation	2.08E-02
GO:0045321	leukocyte activation	2.08E-02
GO:0070161	anchoring junction	2.09E-02
GO:0034599	cellular response to oxidative stress	2.09E-02
GO:0042578	phosphoric ester hydrolase activity	2.13E-02
GO:0005539	glycosaminoglycan binding	2.17E-02
GO:0016628	oxidoreductase activity, acting on the CH-CH group of donors, NAD or NADP as acceptor	2.20E-02
GO:0005179	hormone activity	2.32E-02
GO:0005740	mitochondrial envelope	2.40E-02
GO:0019866	organelle inner membrane	2.40E-02
GO:0000975	regulatory region DNA binding	2.45E-02
GO:0016791	phosphatase activity	2.45E-02
GO:0004672	protein kinase activity	2.46E-02
GO:0045859	regulation of protein kinase activity	2.63E-02
GO:0051219	phosphoprotein binding	2.65E-02
GO:0002763	positive regulation of myeloid leukocyte differentiation	2.68E-02
GO:0031012	extracellular matrix	2.74E-02
GO:0030414	peptidase inhibitor activity	2.76E-02
GO:0040012	regulation of locomotion	2.84E-02
GO:0031099	regeneration	2.85E-02
GO:0008360	regulation of cell shape	2.88E-02
GO:0005743	mitochondrial inner membrane	2.90E-02
GO:0005777	peroxisome	2.90E-02
GO:0031966	mitochondrial membrane	2.90E-02
GO:0045926	negative regulation of growth	2.92E-02
GO:0009063	cellular amino acid catabolic process	2.95E-02
GO:0008083	growth factor activity	2.96E-02
GO:0008284	positive regulation of cell proliferation	2.99E-02
GO:0019915	lipid storage	2.99E-02
GO:0035148	tube formation	3.06E-02
GO:0016788	hydrolase activity, acting on ester bonds	3.25E-02

GO:0046982	protein heterodimerization activity	3.27E-02
GO:0030225	macrophage differentiation	3.33E-02
GO:0031647	regulation of protein stability	3.37E-02
GO:0035295	tube development	3.45E-02
GO:0051251	positive regulation of lymphocyte activation	3.48E-02
GO:0009062	fatty acid catabolic process	3.53E-02
GO:0045664	regulation of neuron differentiation	3.53E-02
GO:0010821	regulation of mitochondrion organization	3.53E-02
GO:0021549	cerebellum development	3.69E-02
GO:0080134	regulation of response to stress	3.73E-02
GO:0009986	cell surface	3.77E-02
GO:0001078	RNA polymerase II core promoter proximal region sequence-specific DNA binding transcription factor activity involved in negative regulation of transcription	3.77E-02
GO:0032504	multicellular organism reproduction	3.89E-02
GO:0008654	phospholipid biosynthetic process	3.92E-02
GO:0042063	gliogenesis	3.96E-02
GO:0019432	triglyceride biosynthetic process	3.99E-02
GO:0042594	response to starvation	3.99E-02
GO:0033674	positive regulation of kinase activity	4.00E-02
GO:0005759	mitochondrial matrix	4.08E-02
GO:0010035	response to inorganic substance	4.16E-02
GO:0030335	positive regulation of cell migration	4.16E-02
GO:0070482	response to oxygen levels	4.16E-02
GO:0042542	response to hydrogen peroxide	4.16E-02
GO:0046460	neutral lipid biosynthetic process	4.20E-02
GO:0001503	ossification	4.20E-02
GO:0016331	morphogenesis of embryonic epithelium	4.20E-02
GO:0007005	mitochondrion organization	4.20E-02
GO:0010001	glial cell differentiation	4.20E-02
GO:0072527	pyrimidine-containing compound metabolic process	4.20E-02
GO:0050821	protein stabilization	4.27E-02
GO:0030890	positive regulation of B cell proliferation	4.29E-02
GO:0055037	recycling endosome	4.34E-02
GO:0005913	cell-cell adherens junction	4.59E-02
GO:0045177	apical part of cell	4.59E-02
GO:0006575	cellular modified amino acid metabolic process	4.59E-02
GO:0044212	transcription regulatory region DNA binding	4.64E-02
GO:0010866	regulation of triglyceride biosynthetic process	4.65E-02
GO:0019395	fatty acid oxidation	4.69E-02
GO:0031076	embryonic camera-type eye development	4.69E-02
GO:2001233	regulation of apoptotic signaling pathway	4.80E-02
GO:0007420	brain development	4.82E-02
GO:0001838	embryonic epithelial tube formation	4.82E-02
GO:0060429	epithelium development	4.82E-02
GO:0030155	regulation of cell adhesion	4.83E-02
GO:2001236	regulation of extrinsic apoptotic signaling pathway	4.89E-02
GO:0050867	positive regulation of cell activation	4.90E-02
GO:0016042	lipid catabolic process	4.96E-02

Table S4. Overlap of PCCL3 PPFP peaks with PPARG peaks in mouse adipocytes and macrophages. The PPARG ChIP-seq data are from [29].

**Sample**	**# of peaks**	**# of peaks ≤10kb to TSS**	**# of peaks with homologs**	**# overlap with PPFP (rat)**
PPFP (rat)	20277	3965	2809	-
PPARG (mouse adipocyte)	2634	870	537	186 (34%)
PPARG (mouse macrophage)	1961	661	411	104 (25%)

P=0.0022, Fisher's exact test, two tailed.

Table S5. Genes in gene sets that are induced by PPFP and repressed by pioglitazone. Three gene sets were induced in the comparison of PPFP cells versus EV cells cultured without pioglitazone, and repressed in the comparison of PPFP cells with pioglitazone versus PPFP cells without pioglitazone. The gene sets are *glutathione metabolism* (KEGG), *peroxidase activity* (GO), and *arachidonic acid metabolism* (KEGG). The 49 unique, significant genes within these gene sets are listed in this table.

**Symbol**	**Description**
Cbr1	carbonyl reductase 1
Cbr3	carbonyl reductase 3
Cyp4a8	cytochrome P450, family 4, subfamily a, polypeptide 8
Cyp4f17	cytochrome P450, family 4, subfamily f, polypeptide 17
Cyp4f5	cytochrome P450, family 4, subfamily f, polypeptide 5
Duox2	dual oxidase 2
Ephx2	epoxide hydrolase 2, cytoplasmic
Gclc	glutamate-cysteine ligase, catalytic subunit
Gclm	glutamate cysteine ligase, modifier subunit
Ggt6	gamma-glutamyl transferase 6
Ggt7	gamma-glutamyltransferase 7
Gpx2	glutathione peroxidase 2
Gpx4	glutathione peroxidase 4
Gpx8	glutathione peroxidase 8
Gsr	glutathione reductase
Gss	glutathione synthetase
Gsta4	glutathione S-transferase mu 2
Gstm2	glutathione S-transferase mu 2
Gstm7	glutathione S-transferase, mu 7
Gsto1	glutathione S-transferase omega 1
Gstp1	glutathione S-transferase pi 1
Gstt1	glutathione S-transferase theta 1
Hpgds	hematopoietic prostaglandin D synthase
Idh1	isocitrate dehydrogenase 1 (NADP+), soluble
Idh2	isocitrate dehydrogenase 2 (NADP+), mitochondrial
Iyd	iodotyrosine deiodinase
LOC501110	similar to Glutathione S-transferase A1 (GTH1) (HA subunit 1) (GST-epsilon) (GSTA1-1) (GST class-alpha)
Lta4h	leukotriene A4 hydrolase
Mgst1	microsomal glutathione S-transferase 1
Mgst2	microsomal glutathione S-transferase 2
Odc1	ornithine decarboxylase 1
Park7	parkinson protein 7
Pla2g2d	phospholipase A2, group IID
Pla2g4a	phospholipase A2, group IVA (cytosolic, calcium-dependent)
Pla2g5	phospholipase A2, group V
Prdx1	peroxiredoxin 1
Prdx2	peroxiredoxin 2
Prdx3	peroxiredoxin 3
Prdx4	peroxiredoxin 4
Prdx6	peroxiredoxin 6
Ptgs1	prostaglandin-endoperoxide synthase 1
Ptgs2	prostaglandin-endoperoxide synthase 2
Rrm1	ribonucleotide reductase M1
Rrm2	ribonucleotide reductase M2
Rrm2b	ribonucleotide reductase M2 B (TP53 inducible)
Sep15	selenoprotein 15
Srm	spermidine synthase
Tpo	thyroid peroxidase
Txndc17	thioredoxin domain containing 17

Table S6. Overlap of PPFP-regulated genes reported in human thyroid carcinomas [5] with PCCL3 PPFP cell RNA-seq and ChIP-seq data

entrezID	symbol	gene name	RNA-seq overlap	ChIP-seq overlap
10060	ABCC9	ATP-binding cassette, sub-family C (CFTR/MRP), member 9	yes	
84836	ABHD14B	abhydrolase domain containing 14B	yes	
30	ACAA1	acetyl-CoA acyltransferase 1	yes	yes
34	ACADM	acyl-CoA dehydrogenase, C-4 to C-12 straight chain	yes	yes
35	ACADS	acyl-CoA dehydrogenase, C-2 to C-3 short chain	yes	yes
80221	ACSF2	acyl-CoA synthetase family member 2	yes	
95	ACY1	aminoacylase 1	yes	yes
56894	AGPAT3	1-acylglycerol-3-phosphate O-acyltransferase 3	yes	yes
51129	ANGPTL4	angiopoietin-like 4	yes	yes
23294	ANKS1A	ankyrin repeat and sterile alpha motif domain containing 1A	yes	
364	AQP7	aquaporin 7	yes	yes
384	ARG2	arginase, type II	yes	yes
84159	ARID5B	AT rich interactive domain 5B (MRF1-like)		yes
443	ASPA	aspartoacylase	yes	yes
445	ASS1	argininosuccinate synthase 1	yes	
598	BCL2L1	BCL2-like 1		yes
79794	C12orf49 (rat RGD1562310)	chromosome 12 open reading frame 49	yes	
57415	C3orf14 (rat RGD1306063)	chromosome 3 open reading frame 14	yes	
9254	CACNA2D2	calcium channel, voltage-dependent, alpha 2/delta subunit 2	yes	
10645	CAMKK2	calcium/calmodulin-dependent protein kinase kinase 2, beta	yes	
84869	CBR4	carbonyl reductase 4	yes	
875	CBS	cystathionine-beta-synthase		yes
56994	CHPT1	choline phosphotransferase 1	yes	
1305	COL13A1	collagen, type XIII, alpha 1	yes	
7852	CXCR4	chemokine (C-X-C motif) receptor 4	yes	yes
57007	CXCR7	atypical chemokine receptor 3	yes	
1628	DBP	D site of albumin promoter (albumin D-box) binding protein	yes	yes
26063	DECR2	2,4-dienoyl CoA reductase 2, peroxisomal		yes
2026	ENO2	enolase 2	yes	
2027	ENO3	enolase 3	yes	
2036	EPB41L1	erythrocyte membrane protein band 4.1-like 1	yes	
64787	EPS8L2	EPS8-like 2	yes	yes
2098	ESD	esterase D	yes	
80004	ESRP2	epithelial splicing regulatory protein 2	yes	
2109	ETFB	electron-transfer-flavoprotein, beta polypeptide		yes
2139	EYA2	eyes absent homolog 2 (Drosophila)	yes	yes
9982	FGFBP1	fibroblast growth factor binding protein 1	yes	yes
2274	FHL2	four and a half LIM domains 2	yes	
10912	GADD45G	growth arrest and DNA-damage-inducible, gamma	yes	yes
2745	GLRX	glutaredoxin (thioltransferase)	yes	yes
2819	GPD1	glycerol-3-phosphate dehydrogenase 1 (soluble)	yes	yes
387509	GPR153	G protein-coupled receptor 153	yes	
2869	GRK5	G protein-coupled receptor kinase 5	yes	
23119	HIC2	hypermethylated in cancer 2	yes	yes
84263	HSDL2	hydroxysteroid dehydrogenase like 2	yes	yes
26034	IPCEF1	interaction protein for cytohesin exchange factors 1	yes	
3708	ITPR1	inositol 1,4,5-trisphosphate receptor, type 1	yes	
23028	KDM1A	lysine (K)-specific demethylase 1A		yes
81606	LBH	limb bud and heart development	yes	yes
57134	MAN1C1	mannosidase, alpha, class 1C, member 1	yes	
129642	MBOAT2	membrane bound O-acyltransferase domain containing 2	yes	
4233	MET	MET proto-oncogene, receptor tyrosine kinase	yes	
9848	MFAP3L	microfibrillar-associated protein 3-like		yes
4259	MGST3	microsomal glutathione S-transferase 3	yes	
58526	MID1IP1	MID1 interacting protein 1	yes	
79083	MLPH	melanophilin	yes	yes
58529	MYOZ1	myozenin 1		yes
4675	NAP1L3	nucleosome assembly protein 1-like 3	yes	
2063	NR2F6	nuclear receptor subfamily 2, group F, member 6		yes

10039	PARP3	poly (ADP-ribose) polymerase family, member 3	yes	
5091	PC	pyruvate carboxylase	yes	
5121	PCP4	Purkinje cell protein 4	yes	
5174	PDZK1	PDZ domain containing 1		yes
5228	PGF	placental growth factor	yes	
5257	PHKB	phosphorylase kinase, beta	yes	
5346	PLIN1	perilipin 1	yes	yes
5376	PMP22	peripheral myelin protein 22	yes	yes
5463	POU6F1	POU class 6 homeobox 1	yes	yes
5468	PPARG	peroxisome proliferator-activated receptor gamma	yes	yes
5578	PRKCA	protein kinase C, alpha		yes
11099	PTPN21	protein tyrosine phosphatase, non-receptor type 21	yes	yes
9232	PTTG1	pituitary tumor-transforming 1	yes	
7837	PXDN	peroxidasin	yes	
55647	RAB20	RAB20, member RAS oncogene family		yes
23108	RAP1GAP2	RAP1 GTPase activating protein 2	yes	
65997	RASL11B	RAS-like, family 11, member B	yes	
83937	RASSF4	Ras association (RalGDS/AF-6) domain family member 4	yes	yes
54715	RBFOX1	RNA binding protein, fox-1 homolog (C. elegans) 1	yes	
388	RHOB	ras homolog family member B		yes
6299	SALL1	spalt-like transcription factor 1	yes	
6338	SCNN1B	sodium channel, non voltage gated 1 beta subunit	yes	
9728	SECISBP2L	SECIS binding protein 2-like		yes
23176	SEPT8	septin 8		yes
23677	SH3BP4	SH3-domain binding protein 4	yes	yes
6573	SLC19A1	solute carrier family 19 (folate transporter), member 1		yes
788	SLC25A20	solute carrier family 25 (carnitine/acylcarnitine translocase), member 20	yes	yes
5172	SLC26A4	solute carrier family 26, member 4		yes
81539	SLC38A1	solute carrier family 38, member 1	yes	yes
80736	SLC44A4	solute carrier family 44, member 4		yes
23428	SLC7A8	solute carrier family 7 (amino acid transporter light chain, L system), member 8	yes	
9748	SLK	STE20-like kinase		yes
8987	STBD1	starch binding domain 1		yes
9900	SV2A	synaptic vesicle glycoprotein 2A	yes	yes
23158	TBC1D9	TBC1 domain family, member 9 (with GRAM domain)	yes	yes
83439	TCF7L1	transcription factor 7-like 1 (T-cell specific, HMG-box)		yes
7942	TFEB	transcription factor EB	yes	
7078	TIMP3	TIMP metallopeptidase inhibitor 3		yes
7086	TKT	transketolase	yes	
7099	TLR4	toll-like receptor 4	yes	
7009	TMBIM6	transmembrane BAX inhibitor motif containing 6	yes	yes
65084	TMEM135	transmembrane protein 135		yes
8742	TNFSF12	tumor necrosis factor (ligand) superfamily, member 12	yes	
55503	TRPV6	transient receptor potential cation channel, subfamily V, member 6		**yes**
7466	WFS1	Wolfram syndrome 1 (wolframin)	yes	yes
7504	XK	X-linked Kx blood group	yes	

Table S7. A set of ROS-related genes is induced in human PPFP follicular carcinomas versus non-PPFP follicular carcinomas.

	PPFP carcinomas expression greater than non-PPFP carcinomas	PPFP carcinomas expression less than non-PPFP carcinomas	Total
Probesets for set of ROS-related genes	35	13	48
All other probesets	9685	10248	19933
Total	9720	10261	19981

P= 0.0007, Fisher's exact test

Three gene sets in our RNA-seq data were induced in PPFP cells versus EV cells without pioglitazone, and repressed in PPFP cells with pioglitazone versus PPFP cells without pioglitazone: *glutathione metabolism* (KEGG), *peroxidase activity* (GO), and *arachidonic acid metabolism* (KEGG). There are 49 unique, differentially expressed genes within these gene sets. We tested whether this set of genes is induced in human PPFP follicular carcinomas versus non-PPFP follicular carcinomas by comparing the expression of all probesets for the 49 genes versus the probesets for all other genes in the human thyroid carcinoma Affymetrix study of Giordano [5].
